# Effectiveness of sleep interventions for rotating night shift workers: a systematic review and meta-analysis

**DOI:** 10.3389/fpubh.2023.1187382

**Published:** 2023-06-22

**Authors:** Bo Min Jeon, Su Hyun Kim, Seung Hwa Shin

**Affiliations:** ^1^College of Nursing, Kyungpook National University, Daegu, Republic of Korea; ^2^College of Nursing, The Research Institute of Nursing Science, Kyungpook National University, Daegu, Republic of Korea; ^3^Department of Nursing, Andong Science College, Andong, Republic of Korea

**Keywords:** shift work schedule, sleep, sleep-wake disorders, systematic review and meta-analysis, sleep interventions

## Abstract

**Background:**

Sleep disturbance is a common issue among rotating night shift workers and is closely related to health risks. The present study aimed to determine the effectiveness of pharmacological and non-pharmacological sleep interventions for the management of sleep disturbance among rotating night shift workers.

**Methods:**

For this systematic review and meta-analysis, we searched six electronic databases—EMBASE, CINAHL, Cochrane Library, PubMed, Scopus, and Web of Science—for randomized controlled trials and clinical trials published from January 1990 to June 2022. The quality of eligible studies was independently assessed by three authors using the Joanna Briggs Institute Critical Appraisal Checklist for randomized controlled trials and quasi-experimental studies. The meta-analysis was performed based on the random effects model using the Comprehensive Meta-Analysis software. The study was conducted following the Preferred Reporting Items for Systematic Reviews and Meta-Analyses guidelines.

**Results:**

Of the 1019 studies retrieved, 30 met the inclusion criteria for the systematic review; 25 were selected for the meta-analysis. Sleep interventions were categorized as follows: pharmacological approach (*n* = 7), light therapy (*n* = 9), cognitive behavioral approach (*n* = 7), aroma or alternative therapy (*n* = 4), and shift schedule modification (*n* = 3). The overall mean effect size of the interventions was moderate (Hedges' *g* = 0.59; 95% confidence interval = 0.33–0.84, z = 4.50, *p* < 0.001).

**Conclusion:**

Sleep interventions were effective in promoting sleep or reducing sleep disturbance among rotating night shift workers. These findings provide evidence of the effectiveness of various pharmacological and non-pharmacological sleep interventions for managing sleep health in the work environment of rotating night shift workers.

## Introduction

Sleep disturbance is one of the chief complaints reported by shift workers with irregular shifts (1). Approximately 15%−20% of the working population in the US and Europe work on shift schedules comprising night shifts (2, 3). In Korea, 82.1% of the nurses in healthcare institutions are working in rotating night shifts (4). A previous study reported higher prevalence rates of insomnia and mental disorders among shift workers than among non-shift workers (5). In particular, night shift and rotating shift workers are highly vulnerable to sleep disturbance because of disrupted endogenous circadian rhythms and sleep–wake cycle (6, 7). Considering that rotating night shift work may increase the risk of chronic diseases, such as cancer, metabolic syndrome, type II diabetes, cardiovascular disease, and gastrointestinal dysfunction, and exacerbate psychomotor vigilance and performance (8–10), identifying and developing the best interventions to promote sleep and prevent adverse effects from rotating night shift work are necessary (11).

In the literature, various types of interventions to reduce sleep disturbances and promote sleep have been evaluated for night shift workers (12–14). These interventions include pharmacological approaches, such as exogenous melatonin, nitrazepam, armodafinil, and caffeine, and non-pharmacological approaches, such as cognitive behavioral therapy, night-shift napping, shift schedule changes, and controlled light exposure (15). However, statistical evidence from meta-analyses supporting these interventions has been limited because of insufficient and poor-quality data (15, 16). Thus, evidence regarding the comparative effectiveness of sleep interventions currently available for rotating night shift workers is lacking. In addition, previous systematic reviews on sleep interventions have not focused on rotating night shift workers but all night shift workers.

To fill this gap, a systematic review and meta-analysis encompassing pharmacological and non-pharmacological approaches for promoting sleep in rotating night shift workers are warranted. Therefore, in the present study, we aimed to examine sleep interventions for rotating night shift workers and evaluate the effectiveness according to the types of intervention. The investigation of sleep interventions in this meta-analysis and systematic review will guide the direction for advancing intervention programs to promote sleep and health in rotating night shift workers.

## Methods

### Study design

The present study was conducted according to the Preferred Reporting Items for Systematic Reviews and Meta-analyses (PRISMA) 2020 statement (17) (Appendix 1).

### Search strategy

Six electronic databases—EMBASE, CINAHL, Cochrane Library, PubMed, Scopus, and Web of Science—were searched for relevant studies. The search keywords included “shift work,” “night work,” “rotating shift,” “sleep,” “insomnia,” “sleep disturbance,” “sleep deprivation,” “sleep problem,” “intervention,” “treatment,” “therapy,” “counseling,” “program,” “CBT,” and/or “self-help.” Appropriate subject headings for each database (i.e., Medical Subject Headings and CINAHL headings) and free-text terms were logically combined using Boolean operators, such as AND, OR, and truncation (Appendix 2). All studies retrieved from database searches were exported to the EndNote X9 citation management software.

### Study selection

The inclusion criteria were as follows: (a) participants were adults (≥18 years) working in rotating night shifts, which are scheduled shifts that change over time including night-shift (00:00–05:00), (b) interventions aimed to promote sleep or improve sleep disturbances among rotating night shift workers, (c) comparators were non-intervention or any other interventions, (d) outcomes included sleep patterns, (e) study designs were randomized controlled trial (RCT) or clinical trial, (f) studies were published between January 1990 and June 2022, and (g) the full texts of studies were published in the English or Korean language. Studies were excluded in case of the following: (a) the intervention was implemented in simulated work environments and (b) the participants were non-shift workers.

**Figure 1:**
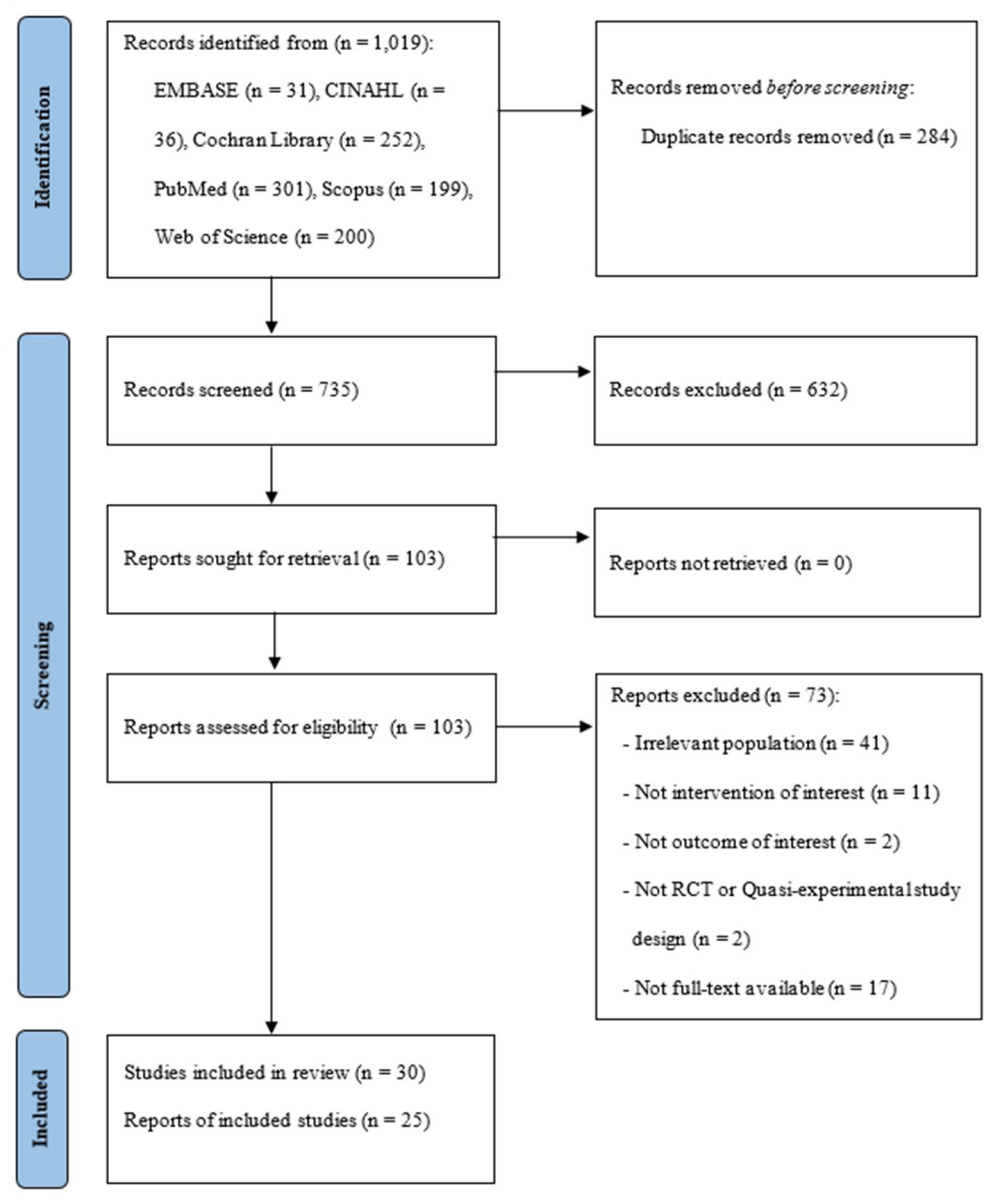
Flow diagram of study selection.

All potentially eligible studies were combined and screened for duplicates. After removing the duplicates, the investigator independently screened the titles and abstracts of each study to determine its eligibility. Three authors assessed the full text of all studies that potentially met the inclusion criteria. Any reasons for exclusion were documented; disagreements were resolved by discussion. The flow diagram of the study is presented in Figure 1.

### Quality assessment

The quality of the included studies was assessed by each investigator independently using the Joanna Briggs Institute Critical Appraisal Checklist for RCTs (13 items) and quasi-experimental studies (9 items) (Appendix 3) (18). The criteria for quality assessment included the following: random sequence generation; allocation concealment; blinding of the participants, personnel, and outcome assessors; incomplete outcome data; selective reporting; and other sources of bias. Overall quality ratings were graded using the categories cited by Reilly et al. (19) (good = at least 80%, moderate = 50%−80%, poor = < 50%).

### Data extraction, synthesis, and analysis

For performing the systematic review, two investigators independently evaluated each study using a data extraction form that comprised the study objective, design, participant characteristics, intervention types, follow-up duration, and outcomes (Appendix 4). The interventions were grouped into similar types, which were finally categorized into five types of interventions: pharmacological approach, light therapy, cognitive behavioral approach, aroma or alternative therapy, and shift schedule modification (Table 1).

**Table 1:** Summarized characteristics of included studies.

**References**	**Study design**	**Sample (*n*)**	**Consecutive days of night shift**	**Types of interventions**	**Sleep measures**
Bjorvatn et al. (20)	Quasi-experimental designs	Oil platform workers (n = 7)	2 weeks	Light	Sleep diary
Bjorvatn et al. (21)	Randomized cross-over	Oil platform workers (*n* = 17)	1 week	Light	Physiologic device
Bozin-Juracić (22)	Quasi-experimental designs	Security workers (*n =* 29)	1 week	Pharmacological	Sleep diary
Budnick et al. (23)	Nonrandomized clinical crossover intervention trial	Industrial workers (*n =* 13)	NR	Light	Sleep logbook
Chang et al. (24)	RCT	Nurses (*n =* 50)	NR	Aroma/Alternative	Physiologic device
Dahlgren et al. (25)	RCT	Nurses (*n =* 207)	NR	CBT/sleep hygiene	Questionnaire
Folkard et al. (26)	RCT	Police officers (n=15)	1 week	Pharmacological	Sleep diary
Franco et al. (27)	Longitudinal intervention study	Nurses (*n =* 17)	≥ 1 day	Aroma/Alternative	Physiologic device
Griepentrog et al. (28)	Randomized cross-over	Nurses (*n =* 43)	NR	Light	Questionnaire
Huang et al. (29)	RCT	Nurses (*n =* 92)	NR	Light	Questionnaire
James et al. (30)	Randomized cross-over	Prehospital personnel (*n =* 22)	4 days	Pharmacological	Sleep diary
Karlson et al. (31)	Longitudinal and controlled field intervention study	Manufacturing workers (*n =* 185)	3 days	Shift schedule change	Questionnaire
Khastar et al. (32)	RCT	Nurses (*n =* 120)	≥ 6 days	CBT/sleep hygiene	Questionnaire
Kim and Hur (33)	RCT	Nurses (*n =* 60)	3 days	Aroma/Alternative	Physiologic device
Kim and Kim (34)	Nonequivalent control grouppre-posttest design	Nurses (*n =* 55)	NR	CBT/sleep hygiene	Questionnaire
Kim (35)	Nonequivalent design with a comparison group	Nurses (*n =* 34)	2 days	Light	Questionnaire
Naimeh et al. (36)	RCT	Midwives (*n =* 30)	NR	Pharmacological	Questionnaire
Niu et al. (37)	RCT	Nursing staffs (*n =* 62)	NR	Shift schedule change	Questionnaire
Niu et al. (38)	RCT	Nurses (*n =* 60)	NR	CBT/sleep hygiene	Physiologic device
Nordin and Knutsson (39)	Quasi-experimental designs	Paper mill workers (*n =* 28)	3–4 days	Shift schedule change	Questionnaire
Pylkkönen et al. (40)	RCT	Truck drivers (*n =* 52)	NR	CBT/sleep hygiene	Physiologic device
Rahman et al. (41)	Randomized cross-over	Nurses (*n =* 9)	2 days	Light	Physiologic device
Sadeghniiat-Haghighi et al. (42)	Randomized cross-over	Nurses (*n =* 86)	NR	Pharmacological	Questionnaire
Sadeghniiat-Haghighi et al. (43)	Randomized cross-over	workers at oil company (*n =* 50)	1 weeks	Pharmacological	Physiologic device
Smith-Coggins et al. (44)	Randomized cross-over	Physicians (*n =* 6)	4–5 days	CBT/sleep hygiene	Physiologic device
Tanaka et al. (14)	Randomized cross-over	Nurses (*n =* 61)	2 days	Light	Questionnaire
van Drongelen et al. (12)	RCT	Airline pilots (*n =* 502)	NR	CBT/sleep hygiene	Questionnaire
Yoon and Song (45)	Repeated measures design	Nurses (*n =* 12)	4 days	Pharmacological	Sleep log
Yoon et al. (46)	Repeated measures cross-over	Nurses (*n =* 12)	4 days	Light	Physiologic device
Zadeh et al. (47)	Single-blind clinical trial	Nurses (*n =* 36)	NR	Aroma/Alternative	Questionnaire

NR, Not reported.

For conducting the meta-analysis, the effectiveness of the intervention was evaluated using the outcome variable of sleep quality; this overarching outcome was calculated by averaging the effect sizes of diverse outcomes associated with sleep reported in each study, such as the total sleep duration, sleep efficiency, subjective sleep quality, and fatigue. These diverse variables were not conceptually different, thereby enabling the use of the average effect sizes within studies (48). Averaging the effect sizes of diverse outcomes in a study precludes the possibility of violating the assumption of data independence and producing imprecise standard errors (SE) and confidence intervals (CIs) from including multiple outcomes of the same participants in the meta-analysis (48).

Studies reporting sufficient statistical data for pooling the effect size calculations were included in the meta-analysis. For the studies measuring sleep quality using more than one method, such as with a diary and questionnaire, the values measured by the questionnaire were used for the meta-analysis. Because the present study aimed to identify the summary effect size of each study, for studies with more than one intervention arm, the sample size of the shared control group was divided by the number of intervention arms to avoid duplicate counting of the participants (49, 50). When the scoring of the scale was not in the same direction, the scores were converted in one direction before synthesis.

In this study, the effect sizes were calculated with Hedges' *g* and 95% CI using the mean values and standard deviations (SDs) of the intervention and control groups reported in the studies. If SDs were not provided, the values were derived using other information (i.e., *p*, t, or F statistics). Hedges' *g* was chosen to reduce the possibility of overestimating the effect size in very small sample sizes (51). Considering the diversity of research methods, samples, intervention types, and outcome measures in each study, a random effects model was used to calculate the summary effects and 95% CI (52). Hedges' *g* can be interpreted as small (0.2), medium (0.5), and large (0.8); a *p*-value of < 0.05 indicated a statistical significance. In the present study, a positive Hedges' *g* indicated that sleep interventions influence the improvement of sleep outcomes.

To investigate the effectiveness of the interventions according to intervention types, preplanned subgroup analysis was used after examining the existence of significant heterogeneity between studies. A subgroup analysis was conducted only in case of a minimum of two studies per subgroup. Heterogeneity between studies was evaluated using the forest plot of the visual test and Higgins *I*^2^ homogeneity test of the quantitative test. *I*^2^ of approximately 25%, 50%, and 75% were interpreted as low, medium, and high heterogeneity, respectively (53).

Publication bias of the included studies was examined using the funnel plot, Egger's test, and Duval and Tweedie's trim-and-fill method. Moreover, sensitivity analysis was performed to evaluate the robustness of the synthesized results. The Comprehensive Meta-Analysis (CMA 3.0) software was used for performing the meta-analysis.

## Results

Overall, 1,019 studies were identified from the database search. Following the removal of 284 duplicates, 103 were screened for full-text review after reviewing titles and abstracts. Finally, 30 studies were included in this study (Figure 1).

### Characteristics of the included studies

A total of 1,972 workers participated in the included studies (Appendix 4). The studies were conducted in 13 countries, including seven European countries, four Asia-Pacific countries, and two North American countries (Appendix 3). Studies with RCT (*n* = 20) and quasi-experimental (*n* = 10) designs were conducted mostly on healthcare workers (*n* = 20, 60.6%). To measure the outcomes, the majority of the studies used subjective tools such as the Pittsburgh Sleep Quality Index (PSQI) and sleep diary (*n* = 20). Other studies utilized actigraphy (*n* = 2) or a combination of objective tools (i.e., polysomnography, actigraphy, and SOMNOwatch) and subjective tools.

Quality assessment in individual studies is summarized in Appendix 3. Among 10 quasi-experimental studies, four studies were determined to be of good quality (31, 34, 35, 46), and the remaining were of moderate quality (20, 22, 23, 27, 39, 45). Among 20 RCTs, only two studies had good quality (38, 44) and 11 studies had moderate quality (12, 14, 21, 24–26, 29, 30, 33, 42); seven studies had poor quality (28, 32, 36, 37, 40, 41, 47) because of allocation concealment (28, 32, 36, 37, 41, 47), blinding of the participants and researchers (28, 32, 36, 37, 40, 41), and insufficient reporting (28, 32, 36, 37, 40, 41, 47).

### Interventions to promote sleep among rotating night shift workers

The interventions to promote sleep among rotating night shift workers were classified into five types: pharmacological approach (*n* = 7), light therapy (*n* = 9), cognitive behavioral approach (*n* = 7), aroma or alternative therapy (*n* = 4), and shift schedule modification (*n* = 3, Table 1, Appendix 3).

First, the pharmacological approach was evaluated in seven studies involving healthcare workers (*n* = 150), industrial or manufacturing workers (*n* = 50), and other occupational workers (*n* = 34, Table 1) (22, 26, 30, 36, 42, 43, 45). With respect to the intervention, melatonin (26, 30, 42, 43, 45), benzodiazepine (22) or non-benzodiazepine (22) class, or *Gingko biloba* (36) was administered as a sleep aid for main sleep (*n* = 7).

Second, light therapy was evaluated in nine studies involving healthcare workers (*n* = 242) and industrial or manufacturing workers (*n* = 37) (14, 20, 21, 23, 28, 29, 35, 41, 46). Interventions include intermittent bright light therapy (14, 20, 23, 28), a combination of bright light and wearing sunglasses (29, 46), wearing glasses fitted with short-wavelength filters (41), comparison of bright light and melatonin (21), and use of an eye shield (35). The intensity of the light interventions ranged from 1,500 to 10,000 lux. The individual exposure duration ranged from 10 min to 10 h; the intervention duration ranged from 4 days to 3 months. The effect of light therapy on sleep enhancement was deemed favorable in all nine studies. The glasses fitted with short-wavelength filters worn for 8 weeks demonstrated a significant improvement in sleep quality and quantity (41).

Third, a cognitive behavioral approach was implemented in seven studies involving healthcare workers (*n* = 388) and other occupational workers (*n* = 554) (12, 25, 32, 34, 38, 40, 44). The interventions included educational programs consisting of sleep physiology, sleep hygiene, and fatigue-relieving and sleep-promoting strategies (*n* = 4) (12, 32, 40, 44), cognitive behavioral interventions (*n* = 2) (25, 34), and exercise interventions (38). The interventions were delivered through face-to-face sessions (25, 32, 40, 44) and mobile technology (12, 34). The mean duration of the interventions was 9 (range, 3–24) weeks. Further, 8 weeks of aerobic exercise showed a significantly positive lasting effect on the total sleep duration measured using actigraphy in 60 female nurses (38).

Fourth, aromatherapy or alternative therapy was evaluated in four studies (24, 27, 33, 47). In two studies evaluating aromatherapy for nurses (24, 33), the interventions using *Lavandula angustifolia* with the inhalation method (33) and *Origanum majorana* with massage therapy (24) showed positive effects on sleep quality. In two other studies of alternative therapy (27, 47), transcutaneous electrical acupoint stimulation (TEAS) on acupuncture points SP6, H7, and LI4 (47) and non-alcoholic beer provided during dinner (27) for nurses positively influenced sleep quality.

Fifth, shift schedule modification was implemented in three studies involving healthcare workers (*n* = 62) and industrial or manufacturing workers (*n* = 213) (31, 37, 39) (Table 1). Interventions involved the modification of the direction of shift rotation (*n* = 2) (31, 37) and shift intervals (*n* = 1) (39).

### Effectiveness of sleep interventions among rotating night shift workers

**Figure 2:**
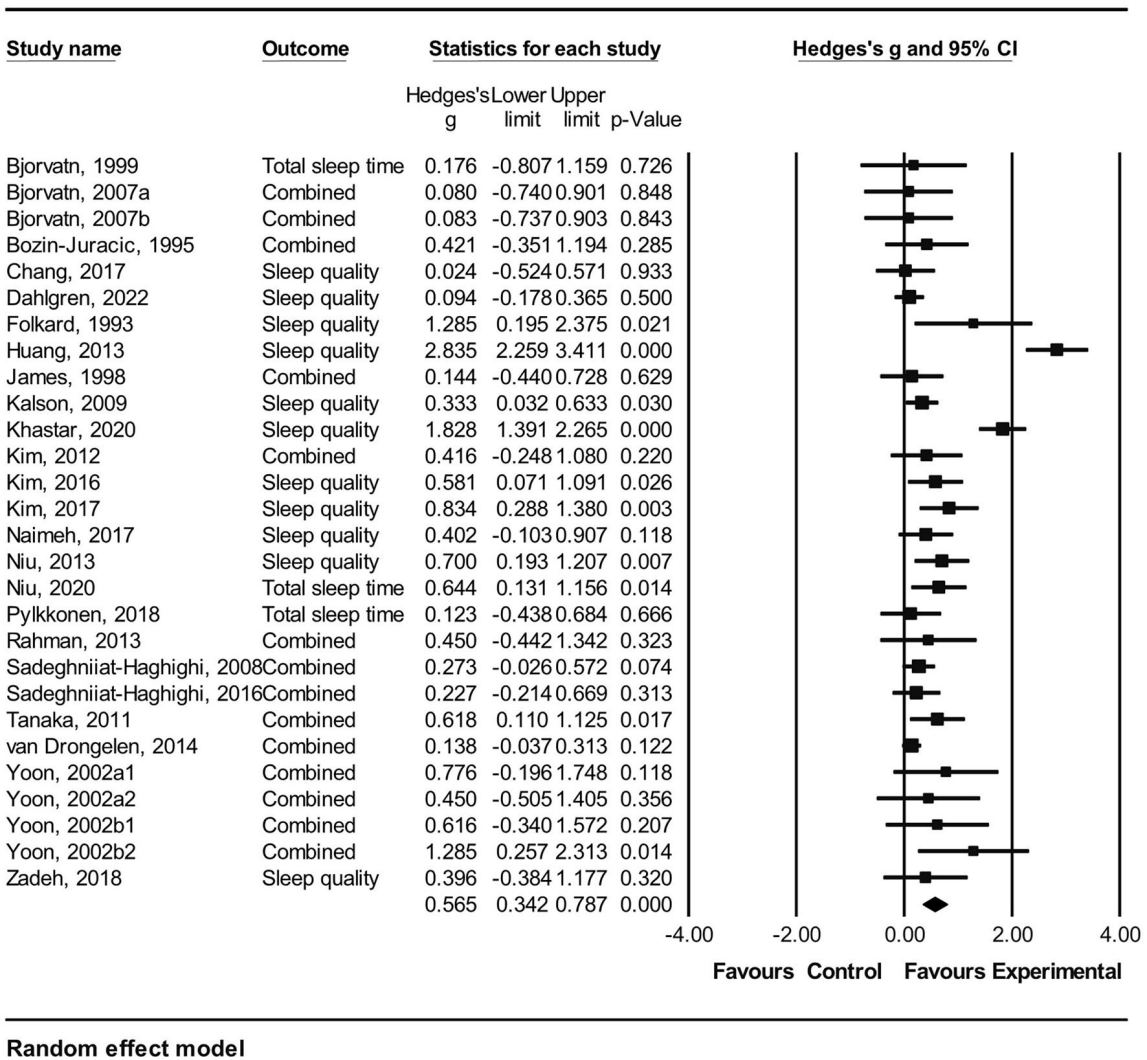
Forest plot for meta analysis of the effect of pharmacological and non-pharmacological sleep interventions among rotating night shift workers. Combined: mean of all reported sleep related outcomes within a study.

Overall, 25 studies were included in the meta-analysis after excluding five studies with insufficient statistical data for pooled effect size calculations (23, 27, 28, 39, 44). The random effect model demonstrated that all intervention types reported a significant effect on the pooled sleep quality, which indicated a moderate effect size (Hedges' *g* = 0.57, 95% CI = 0.34–0.79, *p* < 0.001). Because heterogeneity was high (*I*^2^ = 80.9%, *Q* = 141.26, *p* < 0.001), a subgroup analysis was performed according to the intervention types. The results of the meta-analysis are shown in Table 2 and Figure 2.

**Table 2:** Effectiveness of types of interventions among rotating night shift workers.

**Category**	**Subgroup**	**Studies (*n*)**	**ES (Hedges's *g*)**	**95% CI**	**Z (*p*)**
Types of interventions	Aroma/Alternative therapy	3	0.33	−0.37–1.04	0.92 (0.355)
	Cognitive behavioral approach	6	0.60	0.13–1.16	2.50 (0.012)
	Light therapy	8	0.86	0.39–1.33	3.61 (< 0.001)
	Pharmacological approach	9	0.40	−0.02–0.83	1.86 (0.063)
	Shift schedule modification	2	0.57	−0.30–1.31	1.23 (0.217)

CI, Confidence Interval; ES, Effect Size; Multiple ES for multiple intervention groups.

In the subgroup analysis, light therapy had a large effect size on the pooled sleep quality (Hedges' *g* = 0.86, 95% CI = 0.39–1.33). Moreover, the cognitive behavioral approach showed significant effects on sleep quality (Hedges' *g* = 0.60, 95% CI = 0.13–1.16). However, other interventions—namely, pharmacological approach, shift schedule modification, and aroma or alternative therapy—did not significantly affect the pooled sleep quality (Table 2).

**Figure 3:**
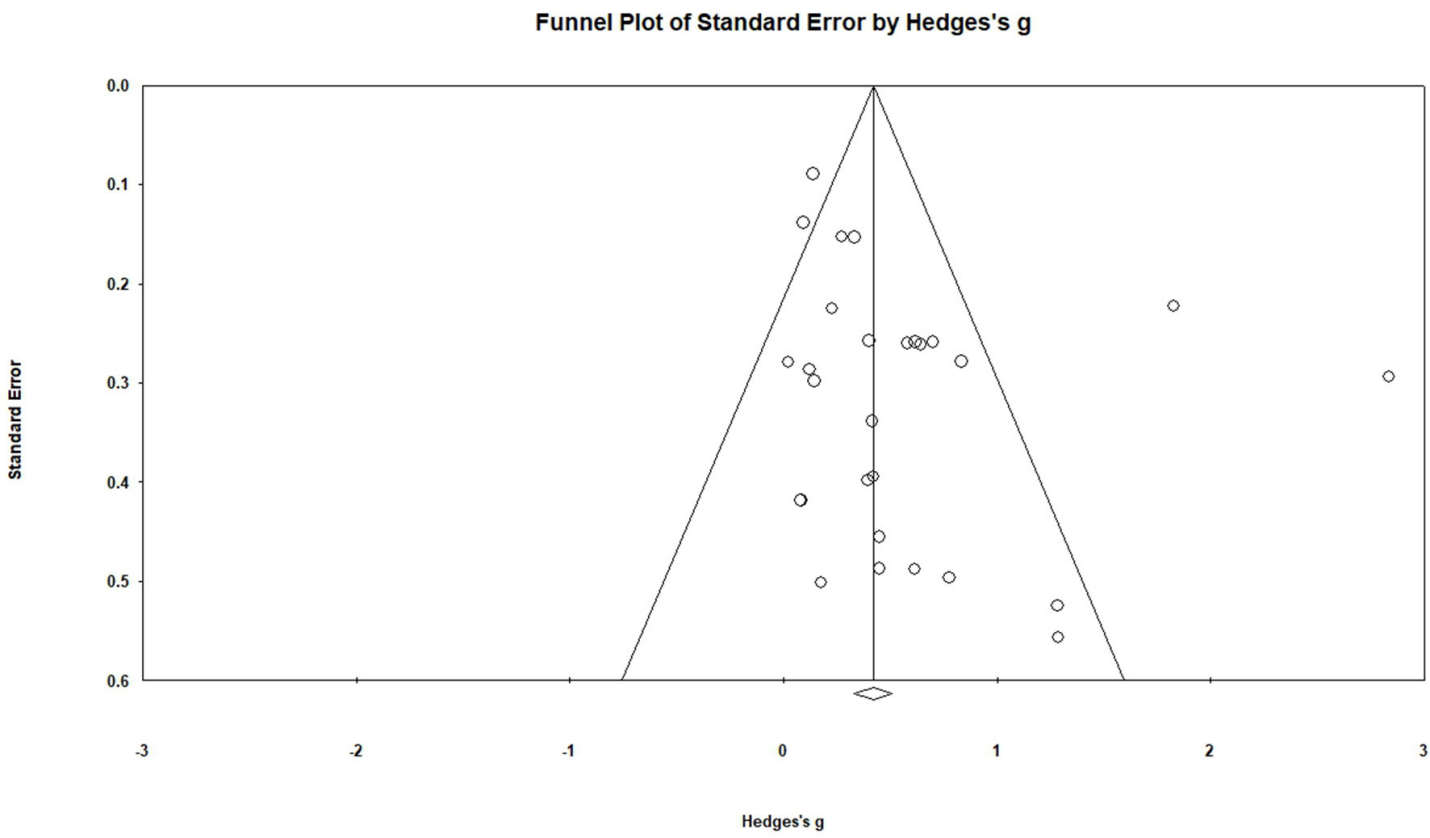
Funnel plots of standard error by Hedges's *g*.

We performed a test of publication bias to identify the validity of the results of the meta-analysis. The funnel plot showed data symmetry. Egger's test showed no significant publication bias (*p* = 0.072) (Figure 3); further, the trim-and-fill analysis showed no change in the effect size. In addition, the sensitivity analysis showed no change in the pooled effect size after excluding the largest weighted study (Hedges' *g* = 0.57, 95% CI = 0.34–0.79, *p* < 0.001) (29). Therefore, the overall effect of these interventions on the combined effects was robust.

## Discussion

In the present systematic review of sleep interventions for rotating night shift workers, five types of interventions were identified: pharmacological approach, light therapy, cognitive behavioral approach, aroma or alternative therapy, and shift schedule modification. The most commonly used intervention type was light therapy, followed by pharmacological and cognitive behavioral approaches. Although the pharmacological approach was the most frequently used approach for rotating night shift workers since the 1990's, a decreasing pattern was observed over time, which might be attributed to concerns regarding safety and dependence on the long-term use of hypnotic agents (54). Rotating night shift workers typically work and sleep at an irregular time. Light exposure suppresses melatonin and helps the circadian phase shift in these workers (14, 55). Moreover, compared with the pharmacological intervention, light therapy has no residual effect and tolerance (56, 57). Therefore, light therapy is an effective intervention for rotating night shift workers because it helps in sleep management related to shift work by inducing melatonin secretion and circadian rhythm adaptation.

Study samples of these five sleep interventions were diverse, ranging from healthcare providers to manufacturing workers who were not assigned to particular types of interventions. The factors that have the highest influence on sleep disturbances might differ according to the characteristics of work and types of occupation (58). For example, extended work, higher workloads, and emotional work demands were factors that influenced sleep disturbances among all types of night shift workers in Korea (59), however, only the influence of emotional work demands and social support and not working hours or higher workloads was significant among night shift working nurses in Korea (60). Therefore, future sleep interventions must be assessed to determine the interventions that provide the best outcomes for each type of worker. In addition, most studies in the present review were of moderate or good quality; however, few RCTs had some limitations related to allocation concealment and blinding of participants, which should be improved (28, 32, 36, 37, 40, 41, 47).

In the present meta-analysis, the significant moderate effects of sleep interventions on sleep outcomes (Hedges' *g* = 0.57) indicated the overall effectiveness of these interventions for improving sleep outcomes among rotating night shift workers. This finding is similar to a previous study on the effects of non-pharmacological interventions on sleep disturbance among shift workers (61); however, a direct comparison was difficult because pharmacological sleep interventions were not analyzed in the previous study. In their review, non-pharmacological interventions among shift workers exerted a substantial effect on sleep duration (Hedges' *g* = 0.73) and a moderate effect on sleep efficiency (Hedges' *g* = 0.48), as measured using objective instruments (61). Therefore, our results provided evidence that sleep interventions could be effectively used to improve sleep outcomes among rotating night shift workers.

Our subgroup analysis demonstrated that light therapy had the largest significant effect on sleep outcomes (Hedges' *g* = 0.86), followed by the cognitive behavioral approach (Hedges' *g* = 0.60). This finding is consistent with previous systematic reviews regarding non-pharmacological interventions, which reported that light therapy is beneficial in improving sleep duration among shift workers (61, 62) because this therapy is a well-known means of shifting the circadian phase (62). In the included studies where light therapy was evaluated, the shift work schedule exhibited a regular pattern with slower rotations and working hours of an average of 12 h per night shift. Additionally, light therapy improved sleep in rotating night shift workers; this finding was consistent with a previous study where sleep parameters of rotating shift workers were effectively improved (61). However, the range of intensity or timing of light exposure was wide across studies (61); therefore, the guidelines for light therapy for rotating night shift workers must be developed and established.

The significant effectiveness of the cognitive behavioral approach in the present study was consistent with the findings of a previous meta-analysis, which showed that cognitive behavioral therapies significantly affect insomnia compared with the control intervention (63, 64). The mechanism underlying the beneficial effect of the cognitive behavioral approach is that this approach promotes the restoration of the sleep mechanism by teaching individual skills to reduce excessive arousal that contributes to insomnia and practicing a lifestyle in harmony with the circadian rhythm of the body (65). These findings reveal that cognitive behavioral therapies are as effective as pharmacological therapy and are the preferred intervention for insomnia (64).

However, despite the pharmacological approach being most frequently used in the literature, this approach did not significantly enhance sleep outcomes. The reason for the non-significant effect size of the pharmacological approach remains unclear; it may be attributed to variability in drug dosage (i.e., melatonin dosage ranges from 3 to 6 mg) and the evaluation method used to determine sleep outcomes. Only 1 of 7 studies involving the pharmacological approach evaluated sleep using objective measurement (i.e., SOMNOwatch).

Furthermore, although the shift schedule modification had a moderate effect size on sleep outcomes (Hedges' *g* = 0.57), it did not significantly improve sleep outcomes. Owing to the small number of studies analyzed in this meta-analysis, further analysis with a sufficient number of studies is warranted to understand the effectiveness of shift schedule modification. The findings may have insufficient power because of the small number of studies for each type of intervention. Further investigation is required to identify the sleep intervention that is more effective for rotating night shift workers.

This study had several limitations. First, the meta-analysis with subgroup analysis did not have sufficient power because of the small number of studies included in the analysis. Second, the diverse methods used to assess sleep outcomes, such as actigraphy, sleep diary, PSQI, and polysomnography, may have contributed to inconsistent or inflated results in studies of the same intervention type (15). To circumvent with this issue, we attempted to pool data under each intervention method to determine the overall effectiveness within each subgroup. Third, subgroup analyses of the intervention effects according to factors such as the duration, timing, or intensity of the intervention, country and year of publication, occupation, and direction of the shift rotation were not performed, which warrants further investigation. Fourth, we performed data search using six databases but not using MEDLINE and PsycINFO and included studies in English or Korean only. Along with limited number of included studies, these methodological limitations of this study require caution about generalization of the results.

Despite these limitations, to the best of our knowledge, this is the first study to conduct a comprehensive quantitative synthesis using up-to-date existing data on various sleep interventions. The findings of the present meta-analysis demonstrated the favorable effects of sleep interventions in rotating night shift workers; however, the intervention that is more effective remains unclear. Although no intervention (pharmacological or non-pharmacological) can restore altered circadian rhythm to baseline levels, adequate sleep management may help reduce negative side effects and improve the quality of life for rotating night shift workers (66). In terms of clinical practice implications, light therapy and cognitive behavioral approach are associated with effectively improving the sleep outcomes among rotating night shift workers, thus providing valuable information for intervention involved in rotating night shift workers' sleep problem. Therefore, light therapy and cognitive behavioral approach should be considered as the important component in the development of interventions to promote sleep health.

## Conclusions

In the literature, sleep interventions for rotating night shift workers were classified into pharmacological approach, light therapy, cognitive behavioral approach, aroma or alternative therapy, and shift schedule modification. The most effective intervention for sleep outcomes was light therapy, followed by a cognitive behavioral approach; however, no significant effectiveness was observed for the pharmacological approach, shift schedule modification, and aroma or alternative therapy for promoting sleep among rotating night shift workers.

Further investigations involving a sufficient number of studies are warranted to compare the effects of each type of intervention and understand the components of interventions for achieving the best outcomes.

## Data availability statement

The original contributions presented in the study are included in the article/Supplementary material, further inquiries can be directed to the corresponding author.

## Author contributions

Study design and manuscript writing: BJ, SK, and SS. Data collection and analysis: BJ and SK. All authors contributed to the article and approved the submitted version.
