# Supplementary material for: Effectiveness of sleep interventions for rotating night shift workers: a systematic review and meta-analysis
Source: Front Public Health. 2023 Jun 22;11:1187382. doi: 10.3389/fpubh.2023.1187382 (PMC10323438; doi:10.3389/fpubh.2023.1187382)
Supplement: Supplementary file 1 [file Table_1.docx]

**APPENDIX 1**

**PRISMA checklists**

| **Section and Topic** | **Item #** | **Checklist item** | **Location where item is reported** |
| --- | --- | --- | --- |
| **TITLE** | | |  |
| Title | 1 | Identify the report as a systematic review. | Title page |
| **ABSTRACT** | | |  |
| Abstract | 2 | See the PRISMA 2020 for Abstracts checklist. | Abstract page |
| **INTRODUCTION** | | |  |
| Rationale | 3 | Describe the rationale for the review in the context of existing knowledge. | Page 1 |
| Objectives | 4 | Provide an explicit statement of the objective(s) or question(s) the review addresses. | Pages 1-2 |
| **METHODS** | | |  |
| Eligibility criteria | 5 | Specify the inclusion and exclusion criteria for the review and how studies were grouped for the syntheses. | Pages 2-3 |
| Information sources | 6 | Specify all databases, registers, websites, organisations, reference lists and other sources searched or consulted to identify studies. Specify the date when each source was last searched or consulted. | Page 2 |
| Search strategy | 7 | Present the full search strategies for all databases, registers and websites, including any filters and limits used. | Appendix 1 |
| Selection process | 8 | Specify the methods used to decide whether a study met the inclusion criteria of the review, including how many reviewers screened each record and each report retrieved, whether they worked independently, and if applicable, details of automation tools used in the process. | Page 3 |
| Data collection process | 9 | Specify the methods used to collect data from reports, including how many reviewers collected data from each report, whether they worked independently, any processes for obtaining or confirming data from study investigators, and if applicable, details of automation tools used in the process. | Page 3-4 |
| Data items | 10a | List and define all outcomes for which data were sought. Specify whether all results that were compatible with each outcome domain in each study were sought (e.g. for all measures, time points, analyses), and if not, the methods used to decide which results to collect. | Page 4 |
|  | 10b | List and define all other variables for which data were sought (e.g. participant and intervention characteristics, funding sources). Describe any assumptions made about any missing or unclear information. | Page 4 |
| Study risk of bias assessment | 11 | Specify the methods used to assess risk of bias in the included studies, including details of the tool(s) used, how many reviewers assessed each study and whether they worked independently, and if applicable, details of automation tools used in the process. | Page 3 |
| Effect measures | 12 | Specify for each outcome the effect measure(s) (e.g. risk ratio, mean difference) used in the synthesis or presentation of results. | Page 4 |
| Synthesis methods | 13a | Describe the processes used to decide which studies were eligible for each synthesis (e.g. tabulating the study intervention characteristics and comparing against the planned groups for each synthesis (item #5)). | Page 3 |
|  | 13b | Describe any methods required to prepare the data for presentation or synthesis, such as handling of missing summary statistics, or data conversions. | Page 4 |
|  | 13c | Describe any methods used to tabulate or visually display results of individual studies and syntheses. | Pages 4-5 |
|  | 13d | Describe any methods used to synthesize results and provide a rationale for the choice(s). If meta-analysis was performed, describe the model(s), method(s) to identify the presence and extent of statistical heterogeneity, and software package(s) used. | Pages 4-5 |
|  | 13e | Describe any methods used to explore possible causes of heterogeneity among study results (e.g. subgroup analysis, meta-regression). | Pages 4-5 |
|  | 13f | Describe any sensitivity analyses conducted to assess robustness of the synthesized results. | Page 5 |
| Reporting bias assessment | 14 | Describe any methods used to assess risk of bias due to missing results in a synthesis (arising from reporting biases). | Page 5 |
| Certainty assessment | 15 | Describe any methods used to assess certainty (or confidence) in the body of evidence for an outcome. | Not reported |
| **RESULTS** | | |  |
| Study selection | 16a | Describe the results of the search and selection process, from the number of records identified in the search to the number of studies included in the review, ideally using a flow diagram. | Page 5 |
|  | 16b | Cite studies that might appear to meet the inclusion criteria, but which were excluded, and explain why they were excluded. | Figure 1 |
| Study characteristics | 17 | Cite each included study and present its characteristics. | Pages 5-7, Table 1, Appendix 3 |
| Risk of bias in studies | 18 | Present assessments of risk of bias for each included study. | Pages 5-7, Appendix 2 |
| Results of individual studies | 19 | For all outcomes, present, for each study: (a) summary statistics for each group (where appropriate) and (b) an effect estimate and its precision (e.g. confidence/credible interval), ideally using structured tables or plots. | Pages 7-8, Table 2, Figure 2 |
| Results of syntheses | 20a | For each synthesis, briefly summarise the characteristics and risk of bias among contributing studies. | Pages 7-8, Table 2, Figure 2 |
|  | 20b | Present results of all statistical syntheses conducted. If meta-analysis was done, present for each the summary estimate and its precision (e.g. confidence/credible interval) and measures of statistical heterogeneity. If comparing groups, describe the direction of the effect. | Pages 7-8, Table 2, Figure 2 |
|  | 20c | Present results of all investigations of possible causes of heterogeneity among study results. | Page 8 |
|  | 20d | Present results of all sensitivity analyses conducted to assess the robustness of the synthesized results. | Page 8 |
| Reporting biases | 21 | Present assessments of risk of bias due to missing results (arising from reporting biases) for each synthesis assessed. | Pages 8, Figure 3 |
| Certainty of evidence | 22 | Present assessments of certainty (or confidence) in the body of evidence for each outcome assessed. | Not reported |
| **DISCUSSION** | | |  |
| Discussion | 23a | Provide a general interpretation of the results in the context of other evidence. | Pages 8-11 |
|  | 23b | Discuss any limitations of the evidence included in the review. | Pages 11-12 |
|  | 23c | Discuss any limitations of the review processes used. | Pages 11-12 |
|  | 23d | Discuss implications of the results for practice, policy, and future research. | Pages 11-12 |
| **OTHER INFORMATION** | | |  |
| Registration and protocol | 24a | Provide registration information for the review, including register name and registration number, or state that the review was not registered. | Not reported |
|  | 24b | Indicate where the review protocol can be accessed, or state that a protocol was not prepared. | Not reported |
|  | 24c | Describe and explain any amendments to information provided at registration or in the protocol. | Not reported |
| Support | 25 | Describe sources of financial or non-financial support for the review, and the role of the funders or sponsors in the review. | Page 12 |
| Competing interests | 26 | Declare any competing interests of review authors. | Page 13 |
| Availability of data, code and other materials | 27 | Report which of the following are publicly available and where they can be found: template data collection forms; data extracted from included studies; data used for all analyses; analytic code; any other materials used in the review. | Not reported |

**APPENDIX 2**

**Search strategy**

| **CINAHL** |
| --- |
| # S25 S6 AND S12 AND S20 AND S24  # S24 S21 OR S22 OR S23  # S23 SU RCT OR TI RCT OR SU RCT  # S22 SU randomized controlled trial OR TI randomized controlled trial OR SU randomized controlled trial  # S21 SU clinical trial OR TI clinical trial OR SU clinical trial  # S20 S13 OR S14 OR S15 OR S16 OR S17 OR S18OR S19  # S19 SU self-help OR TI self-help OR AB self-help  # S18 SU CBT OR TI CBT OR AB CBT  # S17 SU counselling OR TI counselling OR AB counselling  # S16 SU program OR TI program OR AB program  # S15 SU therapy OR TI therapy OR AB therapy  # S14 SU treatment OR TI treatment OR AB treatment  # S13 SU intervention OR TI intervention OR AB intervention  # S12 S7 OR S8 OR S9 OR S10 OR S11  # S11 SU sleep deprivation OR TI sleep deprivation OR AB sleep deprivation  # S10 SU sleep problem OR TI sleep problem OR AB sleep problem  # S09 SU sleep disturbance* OR TI sleep disturbance* OR AB sleep disturbance*  # S08 SU insomnia OR TI insomnia OR AB insomnia  # S07 SU sleep OR TI sleep OR AB sleep  # S06 S1 OR S2 OR S3 OR S4 OR S5  # S05 SU rotating work OR TI rotating work OR AB rotating work  # S04 SU nightwork OR TI nightwork OR AB nightwork  # S03 SU night work OR TI night work OR AB night work  # S02 SU shiftwork OR TI shiftwork OR AB shiftwork  # S01 SU shift work OR TI shift work OR AB shift work |
| **Cochrane Library** |
| # 31 #6 and #14 and #24 and #30 with Cochrane Library publication date Between Jan 1990 and Dec 2020  # 30 #25 or #26 or #27 or #28 or #29  # 29 (RCT):ti,ab,kw  # 28 (randomized controlled trial):ti,ab,kw  # 27 MeSH descriptor: [Randomized Controlled Trial] explode all trees  # 26 (clinical trial):ti,ab,kw  # 25 MeSH descriptor: [Clinical Trial] explode all trees  # 24 #15 or #16 or #17 or 18 or #19 or #20 or #21 or #22 or #23  # 23 (self-help):ti,ab,kw  # 22 (CBT):ti,ab,kw  # 21 (therapy):ti,ab,kw  # 20 MeSH descriptor: [Therapeutics] explode all trees  # 19 MeSH descriptor: [Counseling] explode all trees  # 18 (counseling):ti,ab,kw  # 17 (program):ti,ab,kw  # 16 (treatment):ti,ab,kw  # 15 (intervention):ti,ab,kw  # 14 #7 or #8 or #9 or #10 or #11 or #12 #13  # 13 (insomnia):ti,ab,kw  # 12 (sleep deprivation):ti,ab,kw  # 11 MeSH descriptor: [Sleep Deprivation] explode all trees  # 10 (sleep problem):ti,ab,kw  # 09 (sleep disturbance*):ti,ab,kw  # 08 (sleep):ti,ab,kw  # 07 MeSH descriptor: [Sleep] explode all trees  # 06 #1 or #2 or #3 or #4 or #5  # 05 (rotating work):ti,ab,kw  # 04 (nightwork):ti,ab,kw  # 03 (night work):ti,ab,kw  # 02 (shiftwork):ti,ab,kw  # 01 (shift work):ti,ab,kw |
| **Scopus** |
| (((TITLE-ABS-KEY("shift work")) or (TITLE-ABS-KEY("shiftwork")) or (TITLE-ABS-KEY("night work")) or (TITLE-ABS-KEY("nightwork")) or (TITLE-ABS-KEY("rotating work"))) and ((TITLE-ABS-KEY("sleep")) or (TITLE-ABS-KEY("insomnia")) or (TITLE-ABS-KEY("sleep disturbance*")) or (TITLE-ABS-KEY("sleep problem")) or (TITLE-ABS-KEY("sleep deprivation"))) and ((TITLE-ABS-KEY("intervention")) or (TITLE-ABS-KEY("treatment")) or (TITLE-ABS-KEY("therapy")) or (TITLE-ABS-KEY("program")) or (TITLE-ABS-KEY("counseling")) or (TITLE-ABS-KEY("CBT")) or (TITLE-ABS-KEY("self-help")))) and ((TITLE-ABS-KEY("clinical trial")) or (TITLE-ABS-KEY("randomizedcontrolled trial")) or (TITLE-ABS-KEY("RCT"))) AND (LIMIT-TO ( PUBYEAR,2020) OR LIMIT-TO ( PUBYEAR,2019) OR LIMIT-TO ( PUBYEAR,2018) OR LIMIT-TO ( PUBYEAR,2017) OR LIMIT-TO ( PUBYEAR,2016) ORLIMIT-TO ( PUBYEAR,2015) OR LIMIT-TO ( PUBYEAR,2014) OR LIMIT-TO ( PUBYEAR,2013) OR LIMIT-TO ( PUBYEAR,2012) OR LIMIT-TO ( PUBYEAR,2011) OR LIMIT-TO ( PUBYEAR,2010) OR LIMIT-TO ( PUBYEAR,2009) OR LIMIT-TO ( PUBYEAR,2008)OR LIMIT-TO ( PUBYEAR,2007) OR LIMIT-TO ( PUBYEAR,2006) OR LIMIT-TO ( PUBYEAR,2005) OR LIMIT-TO ( PUBYEAR,2004) OR LIMIT-TO ( PUBYEAR,2003) OR LIMIT-TO ( PUBYEAR,2002) OR LIMIT-TO ( PUBYEAR,2001) OR LIMIT-TO ( PUBYEAR,2000) OR LIMIT-TO ( PUBYEAR,1999) OR LIMIT-TO ( PUBYEAR,1998) OR LIMIT-TO ( PUBYEAR,1997) OR LIMIT-TO ( PUBYEAR,1996) OR LIMIT-TO ( PUBYEAR,1995) OR LIMIT-TO ( PUBYEAR,1994) OR LIMIT-TO ( PUBYEAR,1993) OR LIMIT-TO ( PUBYEAR,1992) OR LIMIT-TO ( PUBYEAR,1991) OR LIMIT-TO ( PUBYEAR,1990) ) AND (LIMIT-TO ( LANGUAGE,"English" ) OR LIMIT-TO ( LANGUAGE,"Korean" ) ) |
| **Web of Science** |
| # 26 #24 AND #20 AND #12 AND #6 Refined by: LANGUAGES: (ENGLISH OR KOREAN)  # 25 #24 AND #20 AND #12 AND #6  # 24 #23 OR #22 OR #21  # 23 TS=randomized controlled trial  # 22 TS=RCT  # 21 TS=clinical trial  # 20 #19 OR #18 OR #17 OR #16 OR #15 OR #14 OR #13  # 19 TS=self-help  # 18 TS=CBT  # 17 TS=counseling  # 16 TS=program  # 15 TS=therapy  # 14 TS=treatment  # 13 TS=intervention  # 12 #11 OR #10 OR #9 OR #8 OR #7  # 11 TS=sleep deprivation  # 10 TS=sleep problem  # 09 TS=sleep disturbance*  # 08 TS=insomnia  # 07 TS=sleep  # 06 #5 OR #4 OR #3 OR #2 OR #1  # 05 TS=rotating work  # 04 TS=night work  # 03 TS=nightwork  # 02 TS=shiftwork  # 01 TS=shift work |
| **PubMed** |
| # 22 (((((((Shift work) OR (Shiftwork)) OR (night work)) OR (nightwork)) OR (rotating work)) AND((((sleep) OR (insomnia)) OR (sleep disturbance*)) OR (sleep deprivation)) OR (sleep problem))) AND (((((((intervention) OR (treatment)) OR (therapy)) OR (counseling)) OR (program)) OR (CBT)) OR (self-help)) Filters: Clinical Trial, Randomized Controlled Trial, English, Korean, from 1990 - 2020 ) AND (((((((Shift work) OR (Shiftwork)) OR (night work)) OR (nightwork)) OR (rotating work)) AND (((((sleep) OR (insomnia)) OR (sleep disturbance*)) OR (sleep deprivation)) OR (sleep problem))) AND (((((((intervention) OR (treatment)) OR (therapy)) OR (counseling)) OR (program)) OR (CBT)) OR (self-help)) Filters: Clinical Trial, Randomized Controlled Trial, English, Korean, from 1990 - 2020)  # 21 ((((((Shift work) OR (Shiftwork)) OR (night work)) OR (nightwork)) OR (rotating work)) AND (((((sleep) OR (insomnia)) OR (sleep disturbance*)) OR (sleep deprivation)) OR (sleep problem))) AND (((((((intervention) OR (treatment)) OR (therapy)) OR (counseling)) OR (program)) OR (CBT)) OR (self-help))  # 20 ((((((intervention) OR (treatment)) OR (therapy)) OR (counseling)) OR (program)) OR (CBT)) OR (self-help)  # 19 self-help  # 18 CBT  # 17 program  # 16 counseling  # 15 therapy  # 14 treatment  # 13 intervention  # 12 ((((sleep) OR (insomnia)) OR (sleep disturbance*)) OR (sleep deprivation)) OR (sleep problem)  # 11 sleep problem  # 10 sleep deprivation  # 09 sleep disturbance*  # 08 insomnia  # 07 sleep  # 06 ((((Shift work) OR (Shiftwork)) OR (night work)) OR (nightwork)) OR (rotating work)  # 05 rotating work  # 04 nightwork  # 03 night work  # 02 Shiftwork  # 01 Shift work |
| **Embase** |

# 25 #6 AND #12 AND #20 AND #24 AND ([english]/lim OR [korean]/lim) AND [1990-2020]/py

# 24 #21 OR #22 OR #23

# 23 'rct':ti,ab,kw

# 22 'randomized controlled trial':ti,ab,kw

# 21 'clinical trial':ti,ab,kw

# 20 #13 OR #14 OR #15 OR #16 OR #17 OR #18 OR #19

# 19 'program':ti,ab,kw

# 18 'self-help':ti,ab,kw

# 17 'cbt':ti,ab,kw

# 16 'counseling':ti,ab,kw

# 15 'therapy':ti,ab,kw

# 14 'treatment':ti,ab,kw

# 13 'intervention':ti,ab,kw

# 12 #7 OR #8 OR #9 OR #10 OR #11

# 11 'sleep deprivation':ti,ab,kw

# 10 'sleep problem':ti,ab,kw

# 09 'sleep disturbance*':ti,ab,kw

# 08 'insomnia':ti,ab,kw

# 07 'sleep':ti,ab,kw

# 06 #1 OR #2 OR #3 OR #4 OR #5

# 05 'rotating work':ti,ab,kw

# 04 'nightwork':ti,ab,kw

# 03 'night work':ti,ab,kw

# 02 'shiftwork':ti,ab,kw

# 01 'shift work':ti,ab,kw

**APPENDIX 3**

**JBI QUASI-EXPERIMENTAL critical appraisal checklist applied for included studies (n= 10)**

| **Author (year)** | **Q1** | **Q2** | **Q3** | **Q4** | **Q5** | **Q6** | **Q7** | **Q8** | **Q9** | **Total (%) / Rating^a^** | |  |
| --- | --- | --- | --- | --- | --- | --- | --- | --- | --- | --- | --- | --- |
| Kim et al., 2017 [26] | Y | Y | Y | Y | Y | Y | Y | Y | Y | | 9/9 (100%)/Good | |
| Kim, 2012 [27] | Y | Y | Y | Y | Y | Y | Y | Y | Y | | 9/9 (100%)/Good | |
| Franco et al., 2012 [30] | Y | Y | U | Y | Y | U | Y | Y | Y | | 7/9 (77%)/Moderate | |
| Karlson et al., 2009 [28] | Y | Y | Y | Y | Y | Y | Y | Y | Y | | 9/9 (100%)/Good | |
| Yoon et al., 2002a [31] | Y | Y | Y | Y | Y | U | Y | N | Y | | 7/9 (77%)/Moderate | |
| Yoon et al., 2002b [29] | Y | Y | Y | Y | Y | Y | Y | Y | Y | | 9/9 (100%)/Good | |
| Nordin et al., 2001 [32] | Y | Y | Y | Y | Y | U | Y | N | N | | 6/9 (66%)/Moderate | |
| Bjorvatn et al., 1999 [33] | Y | Y | N | N | Y | Y | Y | N | Y | | 6/9 (66%)/Moderate | |
| Bozin-Juracić et al., 1996 [34] | Y | Y | N | N | Y | U | Y | N | N | | 6/9 (66%)/Moderate | |
| Budnck et al., 1995 [35] | Y | Y | N | N | Y | Y | Y | N | Y | | 6/9 (66%)/Moderate | |

*Note.* Q1 : Is it clear in the study what is the ‘cause’ and what is the ‘effect’ (i.e. there is no confusion about which variable comes first)?, Q2 : Were the participants included in any comparisons similar?, Q3 : Were the participants included in any comparisons receiving similar treatment/ care, other than the exposure or intervention of interest?, Q4 : Was there a control group?, Q5 : Were there multiple measurements of the outcome both pre and post the intervention /exposure?, Q6 : Was follow up complete and if not, were differences between groups in terms of their follow up adequately described and analyzed?, Q7 : Were the outcomes of participants included in any comparisons measured in the same way?, Q8 : Were outcomes measured in a reliable way?, Q9 : Was appropriate statistical analysis used?; Y : Yes, N : No, U : Unclear; ^a^ Total (%) and ratings : Good - at least 80 %; Moderate - 50-80 %; Poor - less than 50 %(Overall quality was graded using categories cited by Reilly et al. (21))

**JBI RANDOMIZED CONTROLLED TRIALS critical appraisal checklist applied for included studies (n= 20)**

| **Author (year)** | **Q1** | **Q2** | **Q3** | **Q4** | **Q5** | **Q6** | **Q7** | **Q8** | **Q9** | **Q10** | **Q11** | **Q12** | **Q13** | **Total (%) / Rating^a^** |
| --- | --- | --- | --- | --- | --- | --- | --- | --- | --- | --- | --- | --- | --- | --- |
| Dahlgren et al., 2022 [38] | Y | N | Y | N | N | N | Y | Y | N | Y | Y | Y | Y | 8/13 (69%)/Moderate |
| Khastar et al., 2020 [46] | Y | N | Y | N | N | N | U | Y | N | Y | N | Y | N | 5/13 (38%)/Poor |
| Niu et al., 2021 [36] | Y | Y | Y | Y | N | Y | Y | Y | Y | Y | Y | Y | Y | 12/13 (92%)/Good |
| Zadeh et al., 2018 [47] | Y | U | Y | Y | N | N | U | N | N | Y | N | Y | N | 5/13 (38%)/Poor |
| Pylkkonen et al., 2018 [48] | Y | Y | Y | N | N | N | U | Y | N | Y | N | Y | N | 6/13 (46%)/Poor |
| Griepentrog et al., 2018 [49] | Y | N | Y | N | N | N | N | Y | Y | Y | N | Y | N | 6/13 (46%)/Poor |
| Naimeh et al., 2017 [50] | Y | N | Y | N | N | N | N | Y | N | Y | N | Y | N | 5/13 (38%)/Poor |
| Chang et al., 2017 [39] | Y | Y | Y | Y | N | N | Y | Y | N | Y | N | Y | Y | 9/13 (69%)/Moderate |
| Sadeghniiat-Haghighi et al., 2016 [53] | Y | U | Y | Y | Y | N | N | Y | N | Y | N | Y | N | 7/13 (53%)/Moderate |
| Kim et al., 2016 [40] | Y | N | Y | N | N | N | Y | Y | N | Y | Y | Y | Y | 8/13 (69%)/Moderate |
| van Drongelen et al., 2014 [12] | Y | Y | Y | Y | Y | N | N | Y | N | Y | Y | Y | Y | 10/13 (76%)/Moderate |
| Rahman et al., 2013 [51] | Y | U | U | N | N | N | Y | N | N | Y | Y | Y | N | 5/13 (38%)/Poor |
| Niu et al., 2013 [52] | Y | N | Y | N | N | N | N | Y | N | Y | Y | Y | N | 6/13 (46%)/Poor |
| Huang et al., 2013 [41] | Y | N | Y | N | N | N | Y | Y | N | Y | Y | Y | Y | 8/13 (61%)/Moderate |
| Tanaka et al., 2011 [14] | Y | N | Y | N | N | N | Y | Y | Y | Y | N | Y | Y | 8/13 (61%)/Moderate |
| Sadeghniiat-Haghighi et al., 2008 [42] | Y | Y | Y | Y | Y | N | N | Y | N | Y | N | Y | Y | 9/13 (69%)/Moderate |
| Bjorvatn et al., 2007 [43] | Y | Y | Y | Y | Y | N | N | Y | Y | Y | N | Y | Y | 10/13 (76%)/Moderate |
| James et al., 1998 [44] | Y | Y | Y | Y | Y | N | N | Y | N | Y | N | Y | N | 8/13 (61%)/Moderate |
| Smith-Coggins et al., 1997 [37] | Y | Y | Y | Y | Y | N | Y | Y | N | Y | Y | Y | Y | 11/13 (84%)/Good |
| Folkard et al., 1993 [45] | Y | U | Y | Y | Y | N | U | Y | N | Y | N | Y | N | 7/13 (53%)/Moderate |

*Note.* Q1 : Was true randomization used for assignment of participants to treatment groups?, Q2 : Was allocation to treatment groups concealed?, Q3 : Were treatment groups similar at the baseline?, Q4 : Were participants blind to treatment assignment?, Q5 : Were those delivering treatment blind to treatment assignment?, Q6 : Were outcomes assessors blind to treatment assignment?, Q7 : Were treatment groups treated identically other than the intervention of interest?, Q8 : Was follow up complete and if not, were differences between groups in terms of their follow up adequately described and analyzed?, Q9 : Were participants analyzed in the groups to which they were randomized?, Q10 : Were outcomes measured in the same way for treatment groups?, Q11 : Were outcomes measured in a reliable way?, Q12 : Was appropriate statistical analysis used?, Q13 : Was the trial design appropriate, and any deviations from the standard RCT design (individual randomization, parallel groups) accounted for in the conduct and analysis of the trial?; Y : Yes, N : No, U : Unclear; ^a^ Total (%) and ratings : Good - at least 80 %; Moderate - 50-80 %; Poor - less than 50 %(Overall quality was graded using categories cited by Reilly et al. ()

**APPENDIX 4. General characteristics in included studies**

| **Study** | **Population** | **Intervention/Control** | | **Outcomes (Measure)** | **Effect of intervention** |
| --- | --- | --- | --- | --- | --- |
| **Pharmacological therapy** | | | | | |
| Bozin-Juracić et al. 1996 [34] Croatia Quasi-experimental designs | 29 security workers - Working time :8h at night shifts - Type of rotating shifts: Slow - Shift schedules: NR  - Consecutive days of night shift: 1 week | I: 1) 7.5 mg Zopiclone after night shift before main sleep; Three times (n= 9) - 7-day period  2) 5 mg Nitrazepam during a week of night shift; Three times (n= 11)  C: Placebo (n= 9) | (Subjective) 1) Main sleep and all sleeps (Sleep diary) 2) Wakefulness and Tranquility (Bipolar moods and the associated VAS) | | **1. Sleep diary**  The hypnotics groups had a longer total length of main sleep (F = 1.89, *p* < .05) and better efficacy of main sleep (F = 2.09, *p* < .05) and all sleeps during the day (F = 1.86, *p* < .05) than the control group.  **2. Bipolar moods and the associated visual-analogue scale**  No negative effect of hypnotics on the shift worker's mood after waking up. |
| Folkard et al.  1993 [45] UK RCT | 15 police officers on seven successive night shifts - Working time: 8h per day - Type of rotating shifts:  Slow - Shift schedules: Regular  - Consecutive days of night shift: 1week | I: 5 mg Melatonin or Placebo before the day sleep and first four-night sleeps (n = 7)  - 28-day period  C: Baseline (n = 8) | (Subjective) 1) Sleep quality and Sleep Duration (Sleep diary) 2) Mood (Mood checklist) (Objective) 3) Workload (Workload ratings) 4) Midshift performance (Midshift performance measures) | | **1. Sleep diary (Mean ± SE)**  Melatonin resulted in an increase in rated sleep quality (Baseline 50.5 ± 2.54, Melatonin 60.9 ± 2.62 vs. Placebo 50.3 ± 3.19) and in its duration (Baseline 6.91 ± 0.19 h, Melatonin 7.42 ± 0.15 h vs. Placebo 6.98 ± 0.15 h) relative to placebo.  Melatonin improved sleep quality compared to baseline and placebo (*p* < .001) during the 7-night sleeps following the night shift period. No significant effects were found for other sleep outcome measures.  **2. Mood checklist**  Melatonin resulted in a fairly consistent increase in ratings on the alert-tired dimension relative to both the baseline and placebo conditions.  **3. Workload ratings & Mid Shift performance measures**  In letter-target performance tests visual search speed and accuracy were either unchanged or slightly improved. Memory scanning speed and perception of mental load were adversely affected. |
| James et al. 1998 [44] USA Randomized cross-over | 22 prehospital personnel (EMTs or paramedics) - Working time: 8h at night shift - Type of rotating shifts: NR - Shift schedules: NR  - Consecutive days of night shift: 4 days | I: 6mg Melatonin before each of the consecutive day sleeps (n= 22) - 4-day period C: Placebo (n= 22) | (Subjective) 1) Sleep latency, Awakenings during sleep, SE, Duration of daytime naps, and Sleep quality (Sleep diary and Linear visual analogue scale) 2) Mood (Mood checklist) 3) Workload (Workload ratings) | | Supplemental melatonin does not improve sleep quality or duration in EMS personnel working rotating night shifts (*p* > .05). |
| Naimeh et al. 2017 [50] Iran RCT | 30 Midwives - Working time: NR - Type of rotating shifts: NR - Shift schedules: NR | I: G. biloba every 12-h (n= 15) - 30-day period  C: Placebo (n= 15) | (Subjective)  Sleep quality (PSQI) | | **1. PSQI**  No significant difference seen in the total sleep quality following treatment in intervention and control group (*p* = .457). |
| Sadeghniiat-Haghighi et al. 2008 [42] Iran Randomized cross-over | 86 nurses  - Working time: NR - Type of rotating shifts: NR - Shift schedules: NR | I: 5mg Melatonin 30 min before habitual nighttime sleep (n= 86) - 1-day period  C: Placebo (n= 86) | (Subjective) SOL, TST, and NoA (Seven questions: falling asleep, staying asleep and waking up too early) | | **1. Insomnia parameters**  Intervention group was significantly reduced in SOL as compared with both placebo (*p* < .05) and baseline (*p* < .05). No evidence that melatonin altered TST as compared with baseline TST. |
| Sadeghniiat-Haghighi et al. 2016 [53] Iran Randomized cross-over | 50 Tehran Refinary Oil Company rotating shift workers - Working time: NR - Type of rotating shifts: Slow - Shift schedules: Regular  - Consecutive days of night shift: 1week | I: 3 mg Melatonin about 30 min before usual sleep time (n = 25)  - 3-night period  C: Placebo (n = 25) | (Subjective) 1) Difficulty falling asleep (Two measures to assess difficulty falling asleep)  (Objective) 2) TST, SE, SOL, and WASO (Somnowatch) | | **1. Difficulty falling asleep**  103 selected the choices of moderate, severe or very severe on ISI item 1and had sleep onset latency of twenty minutes or more on PSQI item 2.  **2. Somnowatch (Mean ± SD)**  1) SE (%)  I: 85.5 ± 6.3 vs. Baseline: 82.19 ± 9 vs. Placebo: 82.54 ± 8.1, *p* < .05  2) SOL (hours)  I: 0.20 ± 0.15 vs. Baseline: 0.27 ± 0.14 vs. Placebo: 0.31 ± 0.16, *p* < .05 |
| Yoon et al. 2002a [31] Korea repeated measures design | 12 rotating night shift nurses - Working time: 10h per day - Type of rotating shifts: NR - Shift schedules: NR  - Consecutive days of night shift: 4 days | I: 1) 6 mg Melatonin 2nd and 3rd days after night shift with 30min morning sunlight exposure (Mel) (n= 12) - 4-day period  2) 6mg Melatonin 2nd and 3rd days after night shift with wearing dark  sunglasses (Mel-S)  (n= 12)  C: Placebo (Pla) (n= 12) | (Subjective) 1) Sleep quality and Sleep quantity (Sleep log)  2) Nocturnal alertness (VAS) 3) Mood (POMS) (Objective) 4) Performance (DS-CPT) | | **1. Sleep log (Mean ± SD)**  Melatonin groups significantly increased sleep period (*p* = .001) and total sleep time (*p* = .003).  1) Sleep period (min)  Mel: 477.3 ± 58.3 vs. Mel-S: 493.3 ±74.0 vs. Placebo: 421.0 ± 52.2  2) TST (min)  Mel: 436.1 ± 50 vs. Mel-S: 445.9 ± 79.5 vs. Placebo: 380.0 ± 48.3  **2. VAS**  No significant difference was observed among the three treatments (*p* = .158). Nocturnal alertness to be increased by melatonin administration in Mel and Mel-S (*p* = .051, *p* = .259).  **3. POMS & DS-CPT**  No significant differences were observed among the three treatments. |
| **Light therapy** |  |  |  | |  |
| Bjorvatn et al. 1999 [33] Norway Quasi-experimental designs | 7 night workers at oil platform - Working time: 12h per day - Type of rotating shifts: Slow - Shift schedules: Regular  - Consecutive days of night shift: 2 weeks | I: 30-min BL (10,000 lux) exposure at night shift and return home  (n= 7)  - 8-day period  C: No intervention (n=7) | (Subjective) 1) Sleepiness (KSS and ATS)  2) Sleep latency, KSS at bedtime, Ease falling asleep and awakening, Disturbed sleep, WASO, NoA, and TST (Karolinska sleep/wake diary) | | **1. KSS (Means ± SEM)**  Bright light treatment reduced sleepiness significantly (KSS values) at home, whereas during night work the reduction was significant at the 10% level only.  1) Baseline→ 2week night-work period: 4.9 ± 0.1→ 4.7 ± 0.1, *p*= < .10  2) Baseline→ 1 week at home: 5.9 ± 0.1→ 5.2 ± 0.1*, p*= < .01  **2. ATS (Means ± SEM)**  The ATS scale showed similar results to KSS, with bright light treatment causing reductions in sleepiness parameters at home, whereas the effect during night work was at trend level or not significant.  1) Heavy eyelids (Baseline→ 1 week at home): 26.5 ± 3.9→ 12.4 ± 2.7, *p*= < .05  2) Feeling gravel-eyed: 18.3 ± 3.8→ 8.1 ± 2.5, *p*= < .05  3) Quality of day :5.3 ± 0.3→ 4.5 ± 0.2, *p*= < .05  **3. Sleep diary parameters** such as sleep latency, KSS at bedtime, ease falling asleep, disturbed sleep, wake after sleep onset, ease awakening, number of awakenings and total sleep time (time in bed – sleep latency) were unchanged by bright light treatment.  **4. Bright light treatment significantly improved the ratings of both the adaptation period to night work (from 5.0 to 3.1) and the re-adaptation period back to normal circadian rhythm (from 4.9 to 3.2).** Exposure to bright light reduced the number of days for re-adaptation at home from 5.2 to 3.4, whereas during night work the reduction from 3.1 to 2.6 days was not significant. |
| Bjorvatn et al. 2007 [43] Norway Randomized cross-over | 17 night workers at oil rig - Working time: 12h per day - Type of rotating shifts: Slow - Shift schedules: Regular  - Consecutive days of night shift : 1 week | I: Melatonin + BL exposure during the shift (n= 17)  - 3mg Melatonin (1h before bedtime)  - 30-min BL (10,000 lux) exposure  - 14-day period C: Placebo (n= 17) | (Subjective) 1) Sleepiness (KSS & ATS) 2) Subjective sleep efficiency (Sleep diary)  3) Anxiety (HADS)  (Objective) 4) SOL, TST, and SE (Actigraphy) 5) Reaction time (5-minute simple serial reaction-time test) | | **1. KSS (Mean ± SD)**  1) Melatonin gave a significant reduction in sleepiness during the day shift week, when compared with the placebo and bright light. Melatonin 3.9 ± 0.7, Bright light 4.3 ± 1.0, Placebo 4.3 ± 1.1, Compared with placebo and melatonin effect: Effect size = 0.43  **2. ATS (Mean ± SD)**  1) Irresistible sleepiness  Significant difference when compared with the placebo during the day shift. Melatonin 1.6 ± 2.5, Bright light 3.0 ± 3.8, Placebo 3.2 ± 3.8, Compared with placebo and melatonin effect: Effect size = 0.50  2) Fighting sleep  Melatonin 1.8 ± 2.8, Bright light 2.8 ± 2.8, Placebo 3.4 ± 3.5, Compared with placebo and melatonin effect: Effect size = 0.50  3) Quality of day  Melatonin 3.4 ± 0.6, Bright light 3.8 ± 1.0, Placebo 4.1 ± 1.1, Compared with placebo and melatonin effect: Effect size = 0.79  **3. Sleep diary (Mean ± SD)**  SE was significantly higher after the intake of melatonin when compared with after exposure to bright light, whereas there were no significant differences between the placebo and bright light.  1) SOL  Bright light treatment gave a significant reduction when compared with the placebo and melatonin during the night shift.  Melatonin 13 ± 8, Bright light 9 ± 5, Placebo 14 ± 9, Compared with placebo and Bright light effect: Effect size = 0.69  2) TST  Melatonin gave significantly longer total sleep time than the placebo and bright light.  - Night: Melatonin 405 ± 47, Bright light 392 ± 56, Placebo 386 ± 53, Compared with placebo and Melatonin effect: Effect size = 0.38  - Day: Melatonin 355 ± 38, Bright light 318 ± 35, Placebo 340 ± 38, Compared with placebo and Melatonin effect: Effect size = 0.39  3) SE  Melatonin gave significantly higher SE than the bright light treatment during the day shift (*p*= .014).  Melatonin 87 ± 7, Bright light 80 ± 9, Placebo 83 ± 8, Compared with placebo and Melatonin effect: Effect size = 0.53  **4. HADS**  No significant differences between the placebo, melatonin, and bright light conditions.  **5. Actigraphy (Mean ± SD)**  1) SOL  Melatonin significantly increased the SOL when compared with the effect of bright light (*p*= .04) and the placebo (at the trend level) during the day shift.  Melatonin 15 ± 18, Bright light 7 ± 9, Placebo 6 ± 6, Compared with placebo and Melatonin effect: Effect size = -0.67/ Compared with placebo and Bright light effect: Effect size = -0.13  2) TST  Bright light modestly increased total sleep time when compared with the placebo during night shift.  - Night: Melatonin 416 ± 48, Bright light 419 ± 63, Placebo 403 ± 60, Compared with placebo and Melatonin effect: Effect size = 0.24/ Compared with placebo and Bright light effect: Effect size = 0.26  - Day: Melatonin 355 ± 46, Bright light 367 ± 62, Placebo 348 ± 48, Compared with placebo and Melatonin effect: Effect size = 0.15/ Compared with placebo and Bright light effect: Effect size = 0.34  3) SE  Melatonin is nearly significantly different from bright light (*p*= .086) during night shift.  Melatonin 87 ± 8, Bright light 88 ± 8, Placebo 86 ± 9, Compared with placebo and Melatonin effect: Effect size = 0.12/ Compared with placebo and Bright light effect: Effect size = 0.23  **6. Reaction times:** No significant differences between the night shift and day shift. |
| Budnick et al. 1995 [35] USA Nonrandomized clinical crossover intervention trial | 13 industrial workers - Working time: 12h per day - Type of rotating shifts: Slow - Shift schedules: Regular | I: 6-h BL (6,000-12,000 lux) exposure at night shifts (n= 13) - 3-month period  C: No intervention (n= 9) | (Subjective) 1) Alertness (SSS) 2) Sleep patterns and Performance At home (Daily activities logbook)  (Objective) 3) Melatonin levels (Urinary  samples) | | **1. Most findings concerning self-perceived alertness and performance at work, and sleep patterns were mixed and inconsistent**.  **2. Urinary melatonin level**  Significant suppression of Morning melatonin on the night shift, and 50% had a statistically significant circadian change. |
| Griepentrog et al. 2018 [49] USA Randomized cross-over | 43 night shift ICU nurses - Working time: 12h per day - Type of rotating shifts: NR - Shift schedules: NR | I: 10-h BL (1,500-2,000 lux) exposure at night shift (Phase I: n= 26/Phase II: n= 17) - 4-week period  C: No intervention (Phase I: n= 17/ Phase II: n= 26) | (Subjective) 1) Sleepiness (SSS) (Objective) 2) Psychomotor errors (PVT) 3) Melatonin levels (Saliva samples) | | **1. SSS (Mean ± SEM)**  The intervention group significantly reduced subjective sleepiness at the end of the night shift compared to the control group. I: 2.6 ± 0.2 vs. C: 3.0 ± 0.2, Mean difference = -0.4 ± 0.2, *p* = .03  **2. PVT**  The intervention group committed more psychomotor errors than the control group. I: 2.3 ± 0.2 vs. C: 1.7 ± 0.2, Mean difference = 0.6 ± 0.3, *p* = .03  **3. Saliva samples**  No statistically significant. |
| Huang et al. 2013 [41] Taiwan  RCT | 92 rotating shift nurses : E (n = 62)/N (n = 30) - Working time: 8h at night shift - Type of rotating shifts: NR - Shift schedules: NR | I: 30-min BL (7,000-10,000 lux) exposure +Wearing dark sunglasses at daytime (n= 46) - 10-night period  C: No intervention  (n= 61) | (Subjective) 1) Insomnia (ISI) 2) Anxiety and Depression (HADS) | | **1. ISI (Mean ± SD)** Baseline→ after 2weeks  The intervention group showed significant improvement compared with the control group (*p* < .001). After treatment, in the intervention group 37 nurses (80.4%) met the criterion for no insomnia (ISI < 8). I: 17.9 ± 2.5→ 5.7 ± 5.0 vs. C: 17.1 ± 2.3→ 16.9 ± 3.2  **2. HADS**  The intervention group showed significant improvement compared with the control group (*p* < .001). I: 16.2 ± 5.5 → 9.6 ± 3.9 vs. C: 15.1 ± 6.3 → 16.6 ± 5.9 |
| Kim 2012 [27] Korea Nonequivalent design with a comparison group | 34 shift ICU nurses - Working time: NR - Type of rotating shifts: NR - Shift schedules: Irregular  Consecutive days of night shift: 2 days | I: Use of an eye shield (n= 17)  - 2-week period  C: No intervention  (n= 17) | (Subjective) 1) Sleep quality and Sleep quantity (Sleep diary) 2) Depression (K-CES-D)  3) Stress (Perceived stress tool by Lee, 2005) 4) Job satisfaction (Job satisfaction by Kim, 1994) 5) Sleep quality (Quality of sleep tool by Lee, 2004) | | **1. Sleep diary (Mean ± SD)** Baseline→ after 2weeks  Statistically significant differences in awakening frequency after sleep between the two groups (F = 7.24, *p* = .011).  I: 1.76 ± 1.30→ 0.76 ± 1.20 vs. C: 1.29 ± 1.53→ 1.29 ± 1.45  **2. K-CES-D (Mean ± SD)** Baseline→ after 2weeks  There were significant differences between intervention group and control group (F = 8.81, *p* = .006). I: 2.01 ± 0.39→ 1.83 ± 0.34 vs. C: 1.75 ± 0.54→ 1.80 ± 0.48  **3. Perceived stress (Mean ± SD)** Baseline→ after 2weeks  There were significant differences between intervention group and control group (F = 4.90, *p* = .034). I: 2.25 ± 0.47→ 2.09 ± 0.41 vs. C: 2.24 ± 0.58→ 2.24 ± 0.57  **4. Job satisfaction (Mean ± SD)** Baseline→ after 2weeks  There were significant differences between intervention group and control group (F = 4.19, *p* = .049). I: 2.67 ± 0.40→ 2.73 ± 0.40 vs. C: 2.57 ± 0.50→ 2.55 ± 0.49  **5. Sleep quality (Mean ± SD)** Baseline→ after 2weeks  There were significant differences between intervention group and control group (F = 11.27, *p* = .002). I: 2.78 ± 0.36→ 2.94 ± 0.35 vs. C: 2.75 ± 0.50→ 2.76 ± 0.50 |
| Rahman et al.  2013 [51]  Canada  Randomized cross-over | 9 rotating shift nurses  - Working time: 12h per day  - Type of rotating shifts: Slow  - Shift schedules: Regular  Consecutive days of night shift: 2 days | I: Glasses fitted with short-wavelength filters (0% transmission <480 nm) only during night shifts (n= 9)  - 8-week period  C: Standard indoor light (n= 14) | (Subjective)  1) Daily sleep and wake times (Sleep diary)  2) Depression (CES-D)  3) Sleepiness (ESS)  (Objective)  4) Performance (ARES)  5) Day time sleep (PSG)  6) Melatonin levels (Saliva samples) | | **1. Daily sleep diary**  No significant correlation with the magnitude of changes in sleep structure parameters observed under laboratory conditions.  **2. CES-D & ESS**  No significant difference about mood and subjective daytime sleepiness in intervention conditions.  **3. ARES**  1) One-choice reaction task & Go/No-Go test  Reaction time and throughput on vigilance tests were similar to daytime performance under intervention but impaired under baseline on the first night shift (*p* < .01).  2) Subjective sleepiness  Subjective sleepiness increased throughout the night under both baseline (*p* < .01) and intervention conditions (*p* = .01).  **4. PSG**  1) Nighttime sleep (Mean ± SEM)  On the baseline night PSG, TST (*p* < .01) and sleep efficiency (*p* = .01) were significantly decreased and WASO (*p* = .04) were significantly increased in relation to the comparator night sleep.  - TST (min): B 397.67 ± 18.67 vs. C 476.67 ± 17.32  - SE (%): B 78.14 ± 4.01 vs. C 91.42 ± 1.40  - WASO (min): B 73.00 ± 21.43 vs. C 22.42 ± 5.80  Under intervention, TST was increased by a mean of 40 min compared with baseline, WASO was reduced, and SE was increased to levels similar to the comparator night.  - TST (min): I 437.78 ± 14.37 vs. B 397.67 ± 18.67  - SE (%): I 85.94 ±3.32 vs. C 91.42 ± 1.40, *p* = .58  - WASO (min): I 37.39 ± 17.64 vs. C 22.42 ± 5.80, *p* = .91  2) Daytime sleep  Daytime sleep was significantly impaired under both baseline and intervention conditions.  - TST (min): B 307.78 ± 33.16 vs. C 476.67 ± 17.32, *p* < .01; I 341.52 ± 25.86 vs. C 476.67 ± 17.32, *p* < .01  - SE (%): B 68.32 ± 7.07 vs. C 91.42 ± 1.40, *p* = .01; I 75.36 ± 3.99 vs. C 91.42 ± 1.40, *p* = .41  - WASO (min): B 88.72 ± 32.24 vs. C 22.42 ± 5.80, *p* = .03; I 66.67 ± 22.10 vs. C 22.42 ± 5.80, *p* = .11  **5. Salivary melatonin level**  Salivary melatonin levels were significantly higher on the first (*p* < .05) and middle (*p* < .01) night shifts under intervention compared with baseline. |
| Tanaka et al. 2011 [14] Japan Randomized cross-over | 61 nurses - Working time: 16h at night shift - Type of rotating shifts: Rapid - Shift schedules: Irregular  Consecutive days of night shift: 2 days | I: 10-min BL (5,444-8,826 lux) exposure at day shift (n= 61)  - 1-month period  C: No intervention  (n= 47) | (Subjective) 1) Sleepiness (KSS) 2) Self-assessment of night sleep for day shift days (VAS) 3) Fatigue (CIS)  (Objective)  4) Performance (PVT) | | **1. KSS**  Significant improvements were noted in the intervention group compared with the control group for self‐assessed sleepiness at 10:00 [F (1, 55.6) = 9.60; Mean difference = -0.55; 95% CI: -0.91, -0.20*; p* < .001].  **2. VAS**  Significant improvements were noted in the intervention group compared with the control group for self‐assessment of night sleep for day‐shift days [F (1, 116) = 4.9; Mean difference = 0.37; 95% CI: 0.04, 0.70*; p* = .03].  **3. CIS**  Significant improvements were noted in the intervention group compared with the control group for fatigue [F (1, 49.6) = 6.7; Mean difference = -2.13; 95% CI: -3.78, 0.48; *p* = .01].  **4. PVT**  RTs was significantly lower in the intervention group than in the control group [236 msec vs. 264 msec; F (1, 17.0) = 11.0 p < .01], and the reciprocal RTs (1/mean RTs) were also significantly lower in the intervention group than in the control group [F (1,57108) = 7.2, *p*= .01). |
| Yoon et al. 2002b [29] Korea  Repeated measures cross-over design | 12 night shift nurses - Working time: 10h at night shift - Type of rotating shifts: Rapid - Shift schedules: NR  Consecutive days of night shift: 4 days | I: 1) Room Light (RL)  (n= 12)  - Sunlight or 1-h BL (10,000 lux) exposure at next morning  - 4-day period 2) Bright Light (BL)  (n= 12)  - 4-h BL (4,000-6,000 lux) exposure at nighttime - Same morning light exposure as in RL  - 4-day period  3) BL with Sunglasses (BL/S) (n= 12)  - 4-h BL (4,000-6,000 lux) exposure at nighttime + Wearing sunglasses  - 4-day period | (Subjective) 1) Alertness (VAS) (Objective) 2) Sleep quality and Sleep quantity (Actigraphy) 3) Performance (BMT & DSST) | | **1. VAS**  Alertness increased most remarkably in BL/S, followed by BL. VAS of the fourth day of RL, BL, and BL/S were 88.0 ± 18.2, 93.1 ± 17.6, and 109.5 ± 19.6, respectively (*p* < .05, ANOVA). Alertness on VAS in BL/S significantly increased compared to RL (*p* < .05, paired t-test) with no differences between RL and BL or between BL and BL/S.  **2. Actigraphy**  Sleep qualities and quantities were most prominently improved in BL/S, followed by BL.  1) Sleep period time  RL: 299.6 minutes ± 84.2 vs. BL: 321.3 minutes ± 65.8 vs. BL/S: 393.7 minutes ±62.0, respectively (*p* < .05, ANOVA). BL/S was significantly lengthened, compared with that of RL and BL (*p* < .01, *p* < .01, paired t-tests) and sleep period time of BL tended to be longer than that of RL.  2) TST  RL: 275.8 minutes ± 71.8 vs. BL: 308.3 minutes ± 65.0 vs. BL/S: 375.2 minutes ±53.5, respectively (*p* < .05, ANOVA). BL/S was significantly lengthened, compared with that of RL and BL (*p* < .01*, p* < .01, paired t-tests), and BL was longer than that of RL (*p* < .05, paired t-test)  3) Sleep efficiency  RL: 87.5% ± 5.8 vs. BL: 91.4% ± 4.3, vs. BL/S: 93.3% ± 5.5, respectively. RL was significantly lower than that of BL/S and BL (*p* < .01, p < .05, respectively, paired t-tests) |
| **CBT/Sleep hygiene** | |  |  | |  |
| Dahlgren et al.  2022 [38]  Sweden  RCT | 207 newly graduated nurses  - Working time: NR  - Type of rotating shifts: NR  - Shift schedules: NR | I: 2.5h-group-based recovery programme (n= 99)  - 3 session  - Contents: Unwinding from stress, supporting sleep, handling fatigue  - Face-to-Face lecture  - 7-week period  C: No intervention  (n= 108) | (Subjective)  1) Insomnia (ISI)  2) Sleep quality (KSQ)  3) Burn out, fatigue and cognitive weariness (SMBQ)  4) Work induced fatigue (WIPL)  5) Somatic symptoms (SSS8) | | **1. ISI & KSQ (Mean ± SE)** Baseline→ after 6months  Insomnia symptoms and sleep quality showed no significant group by time interaction (*p* = .08 and *p* = .47, respectively).  1) ISI (I: 10.65 ± 0.51→ 8.83 ± 0.59 vs. C: 10.74 ± 0.49→ 10.58 ± 0.54)  2) KSQ (I: 4.07 ± 0.10→ 4.34 ± 0.11 vs. C: 4.03 ± 0.10→ 4.20 ± 0.10)  **2. SMBQ (Mean ± SE)** Baseline→ after 6months  Symptoms of burn out showed significant group by time interactions (I: 3.74 ± 0.12→ 3.66 ± 0.12 vs. C: 3.71 ± 0.11→ 3.84 ± 0.11, *p* = .02).  **3. WIPL (Mean ± SE)** Baseline→ after 6months  Work induced fatigue during free time showed a significant group by time interaction (I: 3.43 ± 0.09→ 3.30 ± 0.11 vs. C: 3.32 ± 0.09→ 3.32 ± 0.10, *p* = .01).  **4. SSS8 (Mean ± SE)** Baseline→ after 6months  Somatic symptoms were relatively stable over time in the intervention group, but the control group reported increased somatic symptoms (I: 10.82 ± 0.61→ 11.96 ± 0.73 vs. C: 11.25 ± 0.58→ 14.69 ± 0.68, *p* = .03). |
| Khastar et al.  2020 [46]  Iran  RCT | 120 rotating nurses  - Working time: NR  - Type of rotating shifts: NR  - Shift schedules: NR  Consecutive days of night shift: ≥ 6 days | I: 90~120- min sleep intervention about sleep quality in female sexual function and scheduling skills (n= 60)  - 3session; Face-to-Face lecture  - give a text message on mobile phone a day  - 3-week period  C: 90-120 min sleep intervention session  (n= 60) | (Subjective)  1) Sleep quality (PSQI)  2) Sexual quality of life (SQOL-F)  3) Ability and efficiency of sexual behaviour (Sexual Self-Efficacy Questionnaire) | | **1. PSQI (Mean ± SD)** Baseline→ after 12weeks  Intervention group showed significant differences in sleep quality compared to the control group [mean difference (CI 99.98%) = −1.89 (−2.40, −1.38)]. I: 7.87 ± 1.79 → 5.24 ± 0.91 vs. C: 7.35 ± 1.64 → 7.13 ± 1.13  **2. SQIL-F & Sexual Self-Efficacy (Mean ± SD)** Baseline→ after 12weeks  Intervention group showed modest increases in sexual self-efficacy [mean difference (CI 99.98%) = 8.82 (6.83, 10.81)] and sexual quality of life [mean difference (CI 99.98%) = 19.64 (18.08, 21.20)].  1) Sexual Self-Efficacy: (I: 98.63 ± 5.45 → 108.33 ± 5.46 vs. C: 98.37 ± 5.18 → 99.25 ± 7.07)  2) SQIL-F: (I: 53.45 ± 4.22 → 72.31 ± 5.36 vs. C: 54.26 ± 2.79 → 53.48 ± 4.47) |
| Kim et al. 2017 [26] Korea Nonequivalent control group pre-posttest design | 55 nurses with rotating three-shift work - Working time: NR - Type of rotating shifts: NR - Shift schedules: NR | I: 60-min group-based CBT-I using mobile SNS per week (n= 25)  - Contents: Sleep diaries & sleep hygiene, stimulus control, sleep restriction, cognitive behaviour therapy, relaxation training  - Once/week (total 8 times)  - 6-week period  C: Sleep education materials using mobile SNS (n= 30) | (Subjective) 1) Dysfunctional beliefs and attitudes about sleep (DBAS-16) 2) Sleep quality (PSQI-K) 3) Sleepiness (ESS)  4) Depression (CES-D) 5) Quality of life (WHOQOL-BREF) | | **1. DBAS-16 (Mean ± SD)** Baseline→ after 6weeks  Intervention group had significantly lower scores on dysfunctional beliefs and attitudes about sleep than control group (I: 83.08 ± 28.80→ 62.40 ± 22.48, Mean difference = -20.68 ± 33.27 vs. C: 80.27 ± 29.56→ 81.77 ± 36.15, Mean difference = 1.50 ± 25.48; t = 2.80; *p* = .007).  **2. PSQI-K (Mean ± SD)** Baseline→ after 6weeks  Intervention group had significantly higher scores on sleep quality than control group (I: 11.80 ± 3.11→ 8.20 ± 3.19, Mean difference = -3.60 ± 3.11 vs. C: 11.77 ± 3.42 → 10.90 ± 3.19, Mean difference = -0.87 ± 2.96; t = 3.34; *p* = .002).  **3. ESS (Mean ± SD)** Baseline→ after 6weeks  Intervention group had significantly lower scores on sleepiness than control group (I: 9.72 ± 5.65 → 6.08 ± 3.79, Mean difference = -3.64 ± 4.87 vs. C: 8.50 ± 4.41→ 7.37 ± 4.90, Mean difference = -1.13 ± 3.41; t = 2.24; *p* = .029).  **4. CESD (Mean ± SD)** Baseline→ after 6weeks  No significant difference between intervention group and control group.  **5. WHOQOL-BREF (Mean ± SD)** Baseline→ after 6weeks  Intervention group had significantly higher scores on quality of life than control group (I: 3.28 ± 0.41→ 3.60 ± 0.44, Mean difference = 0.32 ± 0.46 vs. C: 3.21 ± 0.58→ 3.26 ± 0.66, Mean difference = 0.06 ± 0.36; t = -2.36*; p* = .021). |
| Niu et al.  2021 [36]  Taiwan RCT | 60 female nurses - Working time: 8h per day - Type of rotating shifts: NR - Shift schedules: NR | I: 60-min individual aerobic exercise after day shifts (n= 30)  - 3 times a week for 8 consecutive weeks (total 24 sessions) - Contents: 3 parts; 1) warm-up (15-min), 2) main aerobic exercise (brisk walking combined with jogging; 30-min), 3) relaxation exercise (15-min)  C: Usual activity (n= 30) | (Objective)  TST, SOL, WASO, SE (Actigraph) | | As a result of the 4-week follow up, the positive lasting effect on aerobic exercise was observed only in TST (*p* <.001).  **1. TST (min) (Mean ± SD)**: Baseline→ after 16 weeks  I: 364.80 ± 79.14→ 405.25 ± 73.48 vs. C: 378.46 ± 68.85→ 373.78 ± 64.61 |
| Pylkkonen et al. 2018 [48] Finland RCT | 52 long haul truck drivers - Working time: NR - Type of rotating shifts: NR - Shift schedules: NR | I: 3.5-h group-based alertness management training (n= 31)  - Two parts: 1) 45-min Face to-Face lecture, 2) 1-h workshop  - 1 session; Personalized advice  - 8-week period  C: No intervention  (n= 21) | (Subjective) 1) Sleepiness (KSS and ESS)  2) Subjective sleep and working hours (Sleep log)  (Objective) 3) Total daily sleep (Actigraphy)  4) BMI (BMI) | | No statistically significant intervention-related improvements |
| Smith-Coggins et al. 1997 [37] USA Randomized cross-over | 6 physicians - Working time: 8-9h per day - Type of rotating shifts: Slow - Shift schedules: Regular  - Consecutive days of night shift: 4-5 days | I: 120-min sleep physiology/hygiene education session (n= 3)  - Contents: Sleep physiology, improved shift schedule design, and strategies to maintain alertness  - Face-to-Face lecture; Tailored work schedule during exposure period  - 4-week period  C: No intervention (n= 3) | (Subjective)  1) Subjective sleep, Alertness, and Mood (Subjective logbooks: Daily sleep/wake diary, SSS)  (Objective)  1) Sleep latency, TST, and SE (PSG)  2) Performance (Battery of 3 tests: 1) PVT, 2) ECG, 3) Stimulated intubation using a mannequin) | | **1. Subjective logbooks**  1) Mood rating  : The subjects were more negative during night shifts than they were during day shifts (more sluggish *p* < .04, less motivated *p* < .03, and less clear thinking *p* < .04).  2) Sleep: No significant changes in both groups.  **2. PSG**- Average total sleep time  : After night shifts, the subjects slept significantly less than they did after day shifts (5 hr 13 min vs. 6 hr 20 min; *p* < .05). Total REM time increased from 63.64 min during baseline to 85.38min during the intervention (*p* <.03)  **3. Battery of 3 tests** - Vigilance reaction times and times for intubation: The subjects were significantly slower during night shifts than they were during day shifts (*p* = .007 and *p* < 0.04, respectively), but performances on ECG analysis did not significantly differ between night and day shifts. |
| van Drongelen et al. 2014 [12] Netherland RCT | 502 airline pilots - Working time: NR - Type of rotating shifts: NR - Shift schedules: NR | I: Both the MORE Energy mobile application (app) with tailored advice and a website with background information (n= 251)  - Contents: Advice tailored to flight schedules and personal characteristics, advice regarding the optimal timing of exposure to daylight and the timing and duration of sleep, napping strategies  - 24-week period  C: Guide to websites with standard information about fatigue (Sleep hygiene, working mechanisms of the biological clock)  (n= 251) | (Subjective)  1) Fatigue (Checklist Individual Strength fatigue questionnaire)  2) Need for recovery (Need for Recovery scale from the Dutch Questionnaire)  3) Sleep quality (Jenkins Sleep Scale Subscales of PSQI)  4) Health perception (Dutch version of SF-36)  5) Health-related behaviour (Health related behaviour questionnaire: 1) Self-developed questions on nutritional behaviour, 2) Two questions on the recommended frequency of physical activity and exercise) | | **1. CIS (Mean ± SD)** Baseline→ after 6 months  The intervention group significantly improved fatigue compared to the control group (I: 62.31 ± 21.03→ 59.01 ± 21.19 vs. C: 62.48 ± 22.18→ 62.44 ± 22.81, β= -3.76, 95% CI =-5.81, -1.71, *p* < .001).  **2. Jenkins Sleep Scale**  The intervention group significantly improved sleep quality compared to the control group (I: 7.49 ± 3.86→ 7.29 ± 3.90 vs. C: 7.41 ± 4.05→ 7.78 ± 4.22, β = -0.59, 95% CI = -1.01, -0.17, *p* = .007)  **3. Health related behaviour**  The intervention group significantly improved strenuous physical activity (I: 2.08 ± 1.45→ 2.27 ± 1.43 vs. C: 1.97 ± 1.40→ 1.99 ± 1.36, β = 0.17, 95% CI = 0.02, 0.32, *p* = .028) and snacking behaviour (I: 4.65 ± 3.88→ 3.77 ± 2.38 vs. C: 4.62 ± 3.23→ 4.54 ± 3.09, β = -0.81, 95% CI = -1.26, -0.37, *p* < .001) compared to the control group.  **4. No significant effects were found for other outcome measures.** |
| **Aromatherapy/Alternative** | |  |  | |  |
| Chang et al. 2017 [39] Taiwan RCT | 50 rotating shifts nurses - Working time: NR - Type of rotating shifts: NR - Shift schedules: NR | I: Lay down for 1 hour + music + aromatherapy Massage (n= 27) - 25 min, once/week (total 4times)  - Essential Oils: Origanum majorana (36 %) - Massage Oil Formula: 5 ml of sweet almond oil is combined with 100 μl of sweet marjoram essential oil (2%) - 1-month period  C: Lay down for 1 hour + music (n= 23)  - once/week (total 4 times) | (Subjective)  1) Sleep quality (PSQI)  (Objective)  2) Duration of onset, Duration of deep sleep, Duration of light sleep, Duration of wake/dream time, and Apnea-hypopnea index (ECG detectors: Ezsleep [TX-EK3]) | | **1. PSQI (Mean ± MD)** Baseline → after 4weeks  The intervention group had a significant decrease in total PSQI as compared to before (I: 8.89 ± 2.45→ 7.63 ± 2.22, z = −3.54, *p* < .001). But the control group had no significant difference in the total PSQI.  **2. ECG**  No significant changes were observed in sleep onset time, deep sleep, light sleep, wake/dream time, and AHI between the two groups, whether in the aromatherapy room or at home/dorm. |
| Franco et al. 2012 [30] Spain longitudinal intervention study | 17 nurses rotating and/or night shifts - Working time: NR - Type of rotating shifts: NR - Shift schedules: NR  - Consecutive days of night shift: ≥ 1 day | I: Ingested 330 mL of alcohol-free beer (SanMiguel 0,0% alcohol) with supper (NR)  - 14-day period  C: No intervention (NR) | (Subjective)  1) Anxiety (State Trait Anxiety Inventory)  2) Work stress (ERI model)  (Objective)  3) SE and Total activity (Actigraphy: Actiwatch®) | | **1. StateTrait Anxiety Inventory** (X ± SE)  Anxiety decreased in the intervention group (State Anxiety I: 18.09 ± 3.8 vs C: 20.69 ± 2.14).  **2. Effort Reward Imbalance (ERI) model**  The mean value of the parameter Effort/Reward was 0.93 ± 0.04. Participants were suffering from “stress” as the value was greater than 0.7, indicating a high level of job stress.  **3. Actigraphy (X ± SE)**  Intervention group diminished sleep latency (I: 12.01 ± 1.19 min vs. C: 20.50 ± 4.21 min, *p* ≤ .05) and total activity compared to control group (I: 5284.78 ± 836.99 activity pulses vs. C: 7258.78 ± 898.89 activity pulses, *p* ≤ .05). Sleep efficiency was not significant. |
| Kim et al. 2016 [40] Korea RCT | 60 three shift nurses - Working time: 8h per day - Type of rotating shifts: NR - Shift schedules: NR  Consecutive days of night shift: 3 days | I: Inhale essential oil  (n= 30)  - 3-min, once/day (total 3 times)  - Essential Oils: Lavandula augustifolia  - 3-day period  C: No intervention  (n= 30) | (Subjective)  1) Sleep quality (NRS & VSH)  (Objective)  2) NoA (Actigraph) | | **1. NRS & VSH**  No significant difference in NRS scores between intervention group and control group.  The VSH of the intervention group was higher than the control group (F= 6.39, *p* = .002).  **2. Actigraph**  The NoA between the intervention group and the control group was significantly different after intervention treatment on the 3rd day (F= 13.35, *p* = .001). |
| Zadeh et al. 2018 [47] Iran single-blind clinical trial | 36 shift nurses - Working time: NR - Type of rotating shifts: NR - Shift schedules: NR | I: 1) TEAS of SP6, HT7, LI4 points (real points) (n= 12)  - 5-min, twice/week (total 8 times)  - 1-month period  2) TEAS of points at a distance of 1.5cm from the real points (Sham points group (n= 12)  C: No intervention  (n= 12) | (Subjective)  Sleep quality (PSQI) | | **1. PSQI (Mean ± SD)** Baseline→ after 1 month  Total PSQI Significantly increased in the real points group (12.16 ± 1.46→ 5.25 ± 2.26; *p* < .001) and sham points (11.91 ± 1.62→ 10.50 ± 1.00; *p*= .016). |
| **Shift schedule change** | |  |  | |  |
| Karlson et al. 2009 [28] Sweden longitudinal and controlled field intervention study | 185 manufacturing workers - Working time: 8h per day - Type of rotating shifts: Rapid - Shift schedules: Regular  Consecutive days of night shift: 3 days | I: Slower backward-rotating Schedule shifts (MMM---NNN---AAA---) (n= 118) - 6-month period  C: Daytime work 5 days a week (n= 67) | (Subjective)  1) Sleepiness (KSS)  2) Fatigue and Recovery ability (SOFI)  3) Work-family interference  (Eight items about work-family interference)  4) Subjective health (SRH, SCL-35 and LSHC)  5) Psychosocial workload (JCQ) | | **1. KSS & Swedish Occupational Fatigue Inventory Lack of Energy, Lack of Motivation, and Sleepiness**  The shift workers moderately reduced problems across all these measures compared to the baseline (*p* ≤ .005, ES range = 0.26-0.39).  **2. Single item of time needed for recovery**  57% of the shift workers reported a need for at least 2 days of recovery after a work week with the initial schedule (*p* < .001).  **3. Work-family interference**  The shift worker group displayed a reduced work-to-family interference (*p* < .001, ES = 0.48).  **4. LSHC, Self-Rated Health, and SCL-35**  The shift workers had slightly to moderately improved subjective health and wellbeing at follow-up compared with baseline (*p* range = .001-.02, ES range = 0.17-0.41).  **5. Job Content Questionnaire**  No significantly different development across groups from baseline to follow-up. |
| Niu et al. 2013 [52] Taiwan RCT | 62 nursing staffs - Working time: 8h per day - Type of rotating shifts: NR - Shift schedules: NR | I: Slower forward-rotating Schedule shifts (D→ E→ N)  (n= 32) - 3-month period  C: Fixed-day shifts  (n= 30) | (Subjective)  1) Sleep quality (Sleep diary and CPSQI)  (Objective) 2) Attention (d2 attention test) | | **1. Sleep diaries**  No significant differences between the two groups.  **2. CPSQI**  CPSQI score differed significantly between the two groups, at 7.0 ± 3.3 for the intervention group and 5.0 ± 2.2 for the control group (*p* = .01).  **3. d2 attention test**  There were significant differences between the intervention group and control group in percentage of errors (E %), the total number of items scanned minus error, and concentration performance (CP) scores (*p* = .03, *p*= .02, *p* < .01).  1) E % (Mean ± SD): Phase 3 (3month)  I: 8.6 ± 6.9 vs. C: 4.7 ± 2.6  2) TN-E (Mean ± SD): Phase 3 (3month)  I: 525.4 ± 91.2 vs. C: 573.5 ± 61.4  3) CP (Mean ± SD): Phase 3 (3month)  I: 203.9 ± 57.4 vs. C 240.0 ± 37.4 |
| Nordin et al. 2001 [32] Sweden Quasi-experimental designs | 28 paper mill workers - Working time: 84h per week - Type of rotating shifts: Rapid - Shift schedules: Regular  - Consecutive days of night shift: 3-4 days | I: 84-h 7-week schedule (n= 12) - 9-month period  C: 84h 6-week schedule (n= 16) | (Subjective)  Sleepiness (KSS) | | **1. KSS**  1) Sleepiness at night  : Sleepiness at night was affected by night (F = 4.90, *p* < .05) and hour (F = 33.64*, p* < .001) in both groups.  2) Sleepiness during recovery  : Intervention group was sleepier during the first recovery day compared to the control group (F = 4.02, *p*< .05). Sleepiness was more pronounced on the first recovery day (F = 23.08, *p* < .01). |

*Note.* ARES= Automated Readiness Evaluation System; ATS= Accumulated Time with Sleepiness; BL= Bright light; BMT= Backward masking test; I= Intervention group; C= Control group; CIS= Checklist Individual Strength; D= Day shift; DBAS-16= Dysfunctional Beliefs and Attitudes about Sleep Scale-16; DDST= Digit symbol substitution test; DS-CPT= Degraded-Stimulus Continuous Performance Test; E= Evening shift; N= Night shift; SCL-35= Symptom Checklist–35; CES-D= Center for Epidemiologic Studies Depression Scale; CGI-C= Clinical Global Impressions-Change; CPSQI= Chinese version of the Pittsburgh Sleep Quality Index; GAF= Global Assessment of Functioning; ECG= Electrocardiogram; ERI= Effort Reward Imbalance; ESS= Epworth Sleepiness Scale; FOSQ= Functional Outcomes of Sleep Questionnaire; HADS= Hospital Anxiety and Depression rating scale; ISI= Insomnia Severity Index; JCQ= Job Content Questionnaire; K-CES-D= Korean version of Center for Epidemiologic Studies-Depression; KSS= Karolinska Sleepiness Scale; KSQ= Karolinska Sleep Questionnaire; LSHC= Lund Subjective Health Complaints inventory; NR= Not reported; POMS= Profile of mood states; PSG= Polysomnography; PSQI= Pittsburgh Sleep Quality Index; PSQI-K= Korea Pittsburgh Sleep Quality Index; PVT= Psychomotor vigilance task; SDS-M= Sheehan Disability Scale; SF-36= 36-item Short Form Health Survey; SE= Sleep Efficiency; SMBQ= Shirom-Melamed Burn-out questionnaire; SOFI= Swedish Occupational Fatigue Inventory; SOL= Sleep Onset Latency; SQOL-F= Survey of sexual quality of life-female; SRH= Self-Rated Health; SSS= Stanford Sleepiness Scale; SSS8= Somatic Symptom Scale-8; SWSD= Shift Work Sleep Disorder; TEAS= Transcutaneous Electrical Accupoint Stimulation; TST= Total Sleep Time; NoA= Number of awakening; NRS= Numeric rating scale; VAS= Visual Analogue Scale; VSH= Verran and Synder-Halpern sleep scale; WASO= Wake After Sleep Onset; WHOQOL-BREF = World Health Organization Quality of Life assessment instrument; WIPL= Work Interference with Personal Life index
